# Taxonomy and Biogeography without frontiers – WhatsApp, Facebook and smartphone digital photography let citizen scientists in more remote localities step out of the dark

**DOI:** 10.3897/BDJ.5.e19938

**Published:** 2017-10-10

**Authors:** Nano Suprayitno, Raden Pramesa Narakusumo, Thomas von Rintelen, Lars Hendrich, Michael Balke

**Affiliations:** 1 Nano Tours, Ubud, Indonesia; 2 Museum Zoologicum Bogoriense, Research Center for Biology, Indonesian Institute of Sciences, Cibinong, Indonesia; 3 Museum für Naturkunde, Berlin, Germany; 4 Zoologische Staatssammlung München, Munich, Germany

**Keywords:** Citizen Science, taxonomy, parataxonomists, handphone technology, internet, social media, diving beetles, Bali

## Abstract

**Background:**

Taxonomy and biogeography can benefit from citizen scientists. The use of social networking and open access cooperative publishing can easily connect naturalists even in more remote areas with in-country scientists and institutions, as well as those abroad. This enables taxonomic efforts without frontiers and at the same time adequate benefit sharing measures.

**New information:**

We present new distribution and habitat data for diving beetles of Bali island, Indonesia, as a proof of concept. The species *Hydaticus luczonicus* Aubé, 1838 and *Eretes griseus* (Fabricius, 1781) are reported from Bali for the first time. The total number of Dytiscidae species known from Bali is now 34.

## Introduction

Citizen science (CS) is a currently much promoted and well-funded approach that seeks to stimulate the public to support scientists and *vice versa* (e.g. www.citizen-science-germany.de; www.buergerschaffenwissen.de). CS has different origins based on different concepts that date back until the mid-nineties, and we here refer to Riesch and Potter (2013) for a short review. We opted to follow the definition provided by the CS Central website operated by the Cornell University Ornithology Lab, which we feel comes closest to our perception of and needs from CS: CS is '*projects in which volunteers partner with scientists to answer real-world questions*' (Citizen Science Central 2017). We would, however, add that Citizen Scientists can very well gain extensive experience and expertise through training, participation and encouragement. In the field of biology, this could, for example, eventually lead to the training of parataxonomists and/or paraecologists, which has proven to be a highly productive approach from the social / cultural interaction all the way to a highly productive scientific level (Basset et al. 2000, Novotny et al. 2012).

However, of the data generated by the "usual" CS projects, only a few (12% out of 388 projects) are being published in peer-reviewed scientific articles (Theobald et al. 2015). This should be a strong incentive to create a platform that can connect professional scientists and citizen science in order to create more sustainable outcomes.

In a way, many taxonomists always were and still are highly advanced citizen scientists, i.e. extremely motivated amateurs educating themselves and often growing into world-leading authorities in their particular field. And recently, there has been increasing cooperation and exchange between such taxonomists and "professional" scientists (i.e. those whose job description includes taxonomy and systematics etc.). An example is DNA barcoding projects where museum scientists rely heavily on the expertise of amateur taxonomists who are often the only ones with knowledge of the taxonomy and ecology of certain taxa. Reassuringly, this has also led to co-authored publications (e.g. Rulik et al. (2017)). But these are experiments that were made in central Europe where communication among scientists and other stakeholders is usually well developed and access to taxonomic knowledge is comparably easy due to the existence of significant historical library and collection resources.

However, most of the world’s biodiversity is not in central Europe, but in tropical countries where it is not always easy, even for professional taxonomists, to access data and exchange ideas, let alone citizens access adequate resources.

At the same time, internet utilization is growing worldwide. Particularly in developing countries, in 2015 the adult internet user and mobile technology ownership reached 54% and 37% of the total population respectively and social networking usage is likely to be higher than in developed countries (Pousther 2016). Therefore, information accumulation and exchange around the globe becomes easier and faster. This new internet technology can be used by taxonomists and other interested citizens for educational and scientific purposes and this is the scope of the present study.

**Figure 1.**
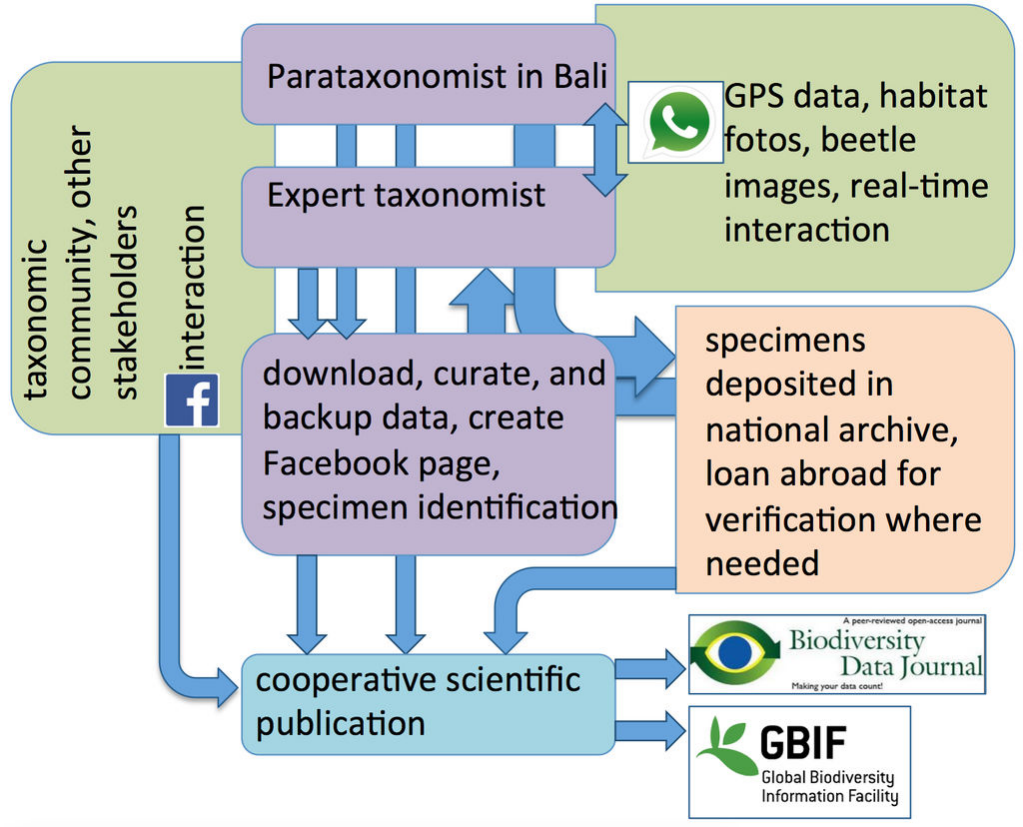
Workflow for this and especially future projects as proposed in this study.

Here, we describe a simple approach (Fig. 1) to cooperate with a citizen scientist. In this case, in Bali, Indonesia, with the goal to conduct a sustainable, scientific experiment using (1) simple, existing technology and (2) enable this citizen scientist to actively participate in the experiment, at the same time (3) building capacity and (4) thus enable this citizen scientist to become part of an interactive scientific community and develop taxonomic skills. That citizen scientist is the tourist operator Suprayitno, the first author of this paper, who discovered his interest in entomology in 2016 as a simple pastime, being a fully independent and self-funded hobbyist. He established contact with the international entomological community via a Facebook group and thereby got attracted strongly to aquatic insects. Discussions and specimen identifications were made online on the social media site Facebook and via WhatsApp, which prove a powerful tool to exchange ideas and especially photographs of sufficient quality in order to help the citizen scientist to expand his taxonomic knowledge quickly. At the same time, discussion partners generated awareness of the CS that specimens and data should be stored in a sustainable manner, leading to the donation of his samples to the national depository of zoology, the Museum Zoologicum Bogoriense, which now houses the entire aquatic beetle collection from Bali. Thus, a pastime was strongly and quickly developed into a serious scientific activity with the aid of social media. This led to a CS project, to the development of taxonomic skills and to objective contributions to a large national museum collection.

This is the focus of our study. We demonstrate the feasibility of our approach by providing empirical data on the biogeography (faunistics) and taxonomy of selected Balinese aquatic Coleoptera because the fauna of Bali is comparably well known and the species selected for our study can be identified based on photographs taken with a handphone camera. The longer-term project goal is an extensive inventory of the Balinese fauna, for which we will propose a collaborative project in the future.

## Materials and methods

### Hardware

We use an Indonesian made Polytron handphone 4G450. Habitat images were taken with the built-in camera (5MP, 2592 x 1944 pixels). The Geo-tagging function was used to map sampling localities in Google Maps.

For detailed photography of specimens, this camera was enhanced with clip-on magnification lenses according to beetle size, i.e. a 2.8X "macro lens" (article DCK004649) and a 20X "super macro/microscope lens" (article DCK004647) from dckina.com (obtained for approx. 12US$ each).

### Workflow

The citizen scientist (in Fig. 1 referred to as parataxonomist) is in Bali and conducts fieldwork during his spare time. Specimens are stored in Ethanol and labeled according to locality, each with a unique collecting event identifier (e.g. BALI_NS_2016_09 which translates to ISLAND_COLLECTOR(NanoSuprayitno)_YEAR_LocalityNumber). These data are shared with collaborators by WhatsApp. Project partners translate the data into Darwin Core format for publication and GBIF upload. Photos of the sampling site and a Google Maps "dropped pin" URL with the GPS coordinates are sent via WhatsApp to a collaborator abroad as a backup and for further databasing. Digital images of beetles and habitats were also disseminated to a wider community of entomologists and other CS via a Facebook project page (https://www.facebook.com/Baliwaterbeetles/), and then species pages will be created for each species separately. In the course of this experiment, the CS was provided with a laptop to empower him to pursue the databasing work independently.

In general, some species can be identified based on photos. Merely using photographs, others can only be identified to genus, or species group. Many other species need to be examined by an expert and usually be dissected for proper identification. Thus, future investigation of the collected and documented specimens might be desirable. Our voucher specimens were, therefore, presented to a national repository, in this case the Indonesian Institute of Sciences, Zoological Museum: Museum Zoologicum Bogoriense (LIPI/MZB). From here, specimens can be shipped abroad as loans for taxonomic identification via standard inter-museum loan procedures.

The result of such a cooperative effort is scientific publications of new data gained as well as an improved national collection.

## Data resources

### Handphone digital imaging resources

Here, we provide the original images taken with a handphone camera and publicized via WhatsApp and Facebook as the starting point of the present project.

**Figure 2.**
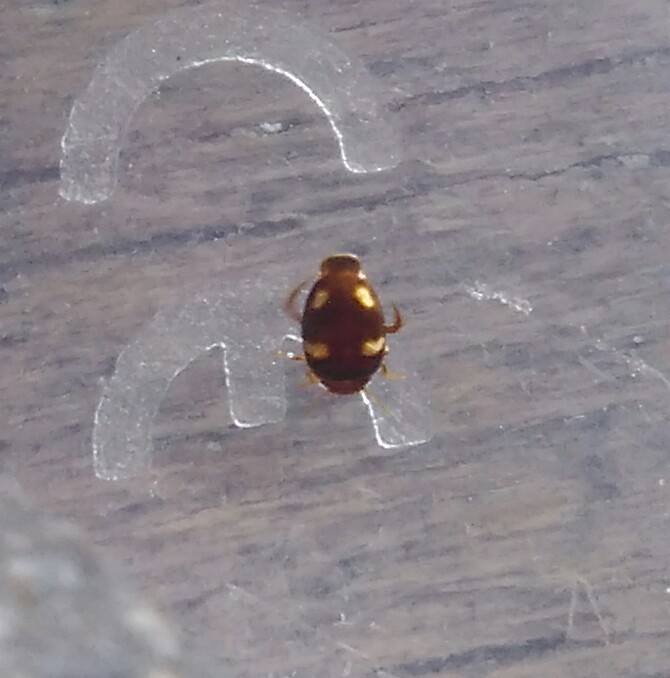
*Allopachria quadripustulata*, HP image from BALI_NS_2016_09.

*Allopachria quadripustulata* (*Fig. 2*).

**Figure 3.**
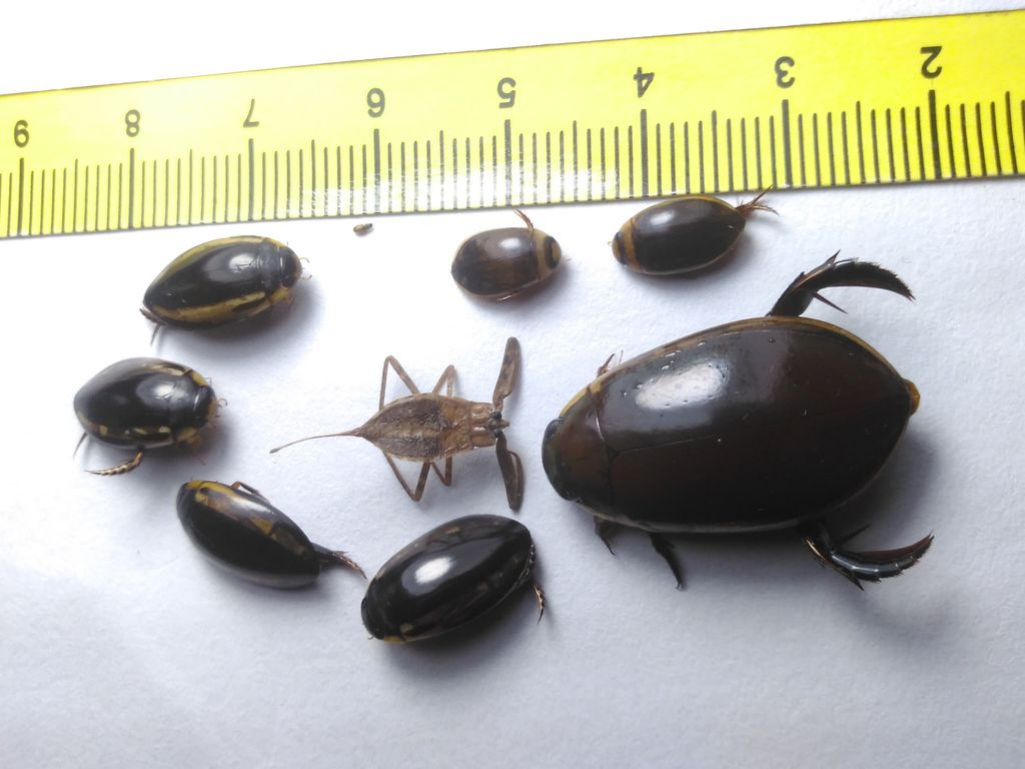
Dytiscidae image taken with handphone camera BALI_NS_2016_05 (large specimen: *Cybister tripunctatus temnenkii*; two smaller beetles above the *Cybister*: *Hydaticus fabricii*; four specimens on the left: *Hydaticus bipunctatus conjungens*).

**Figure 4.**
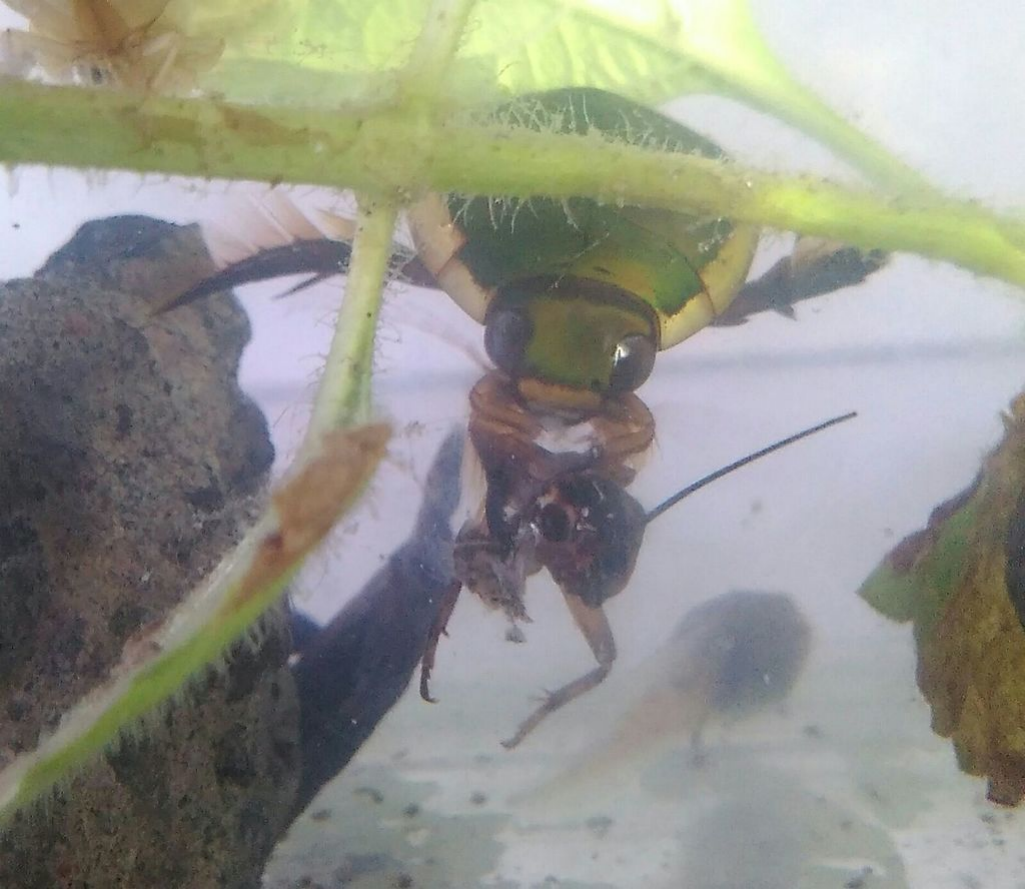
*Cybister tripunctatus*, HP image from Bali.

*Cybister tripunctatus* (*Figs 3, 4*)

**Figure 5.**
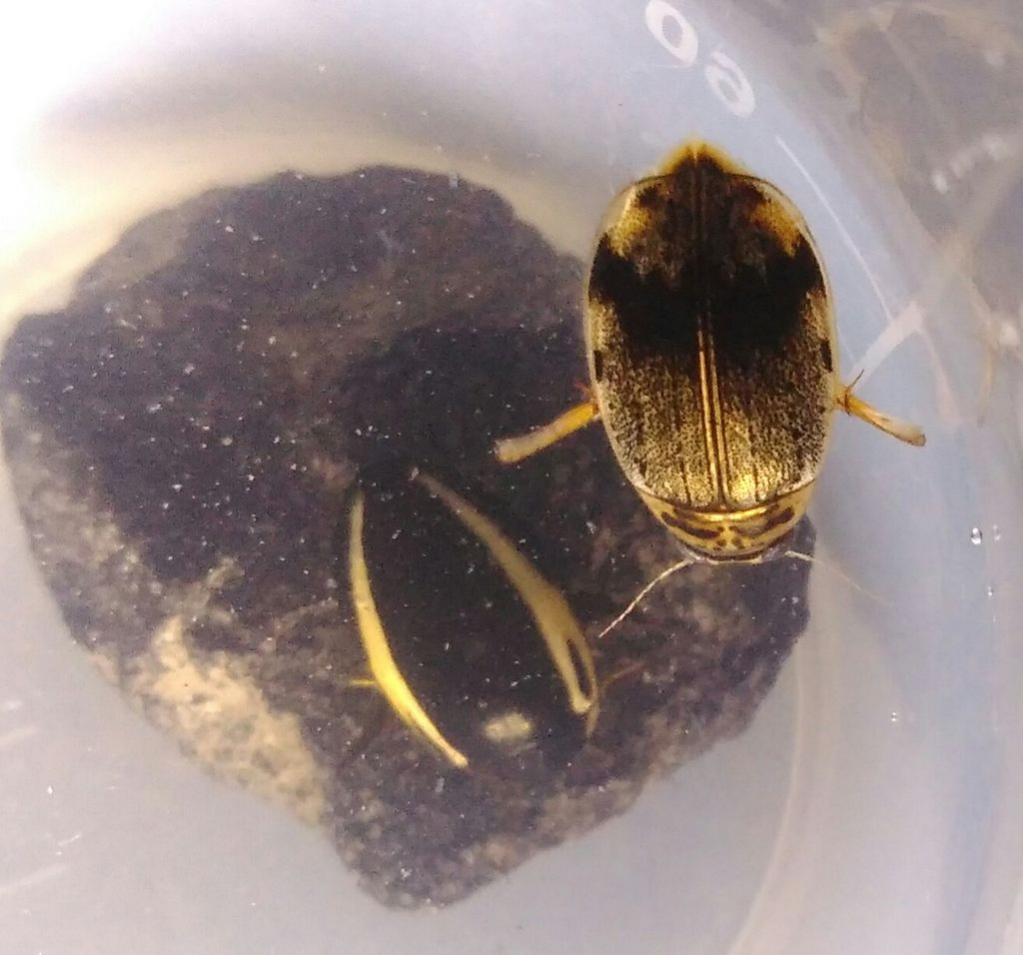
*Eretes griseus*, HP image from BALI_NS_2016_19 (background: *Hydaticus bipunctatus conjungens*).

**Figure 6.**
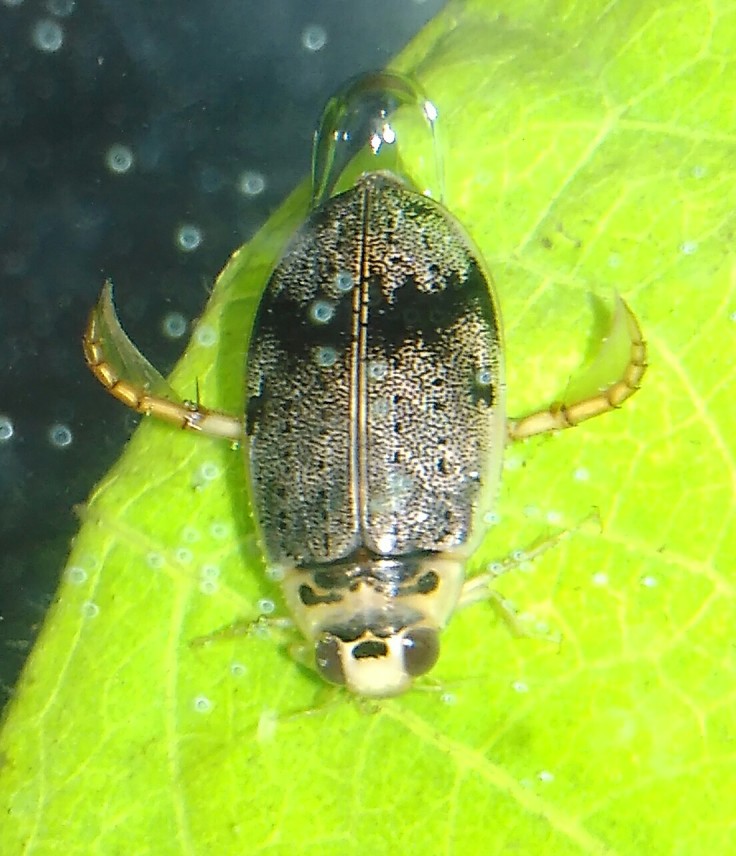
*Eretes griseus*, HP image from BALI_NS_2016_34.

**Figure 7.**
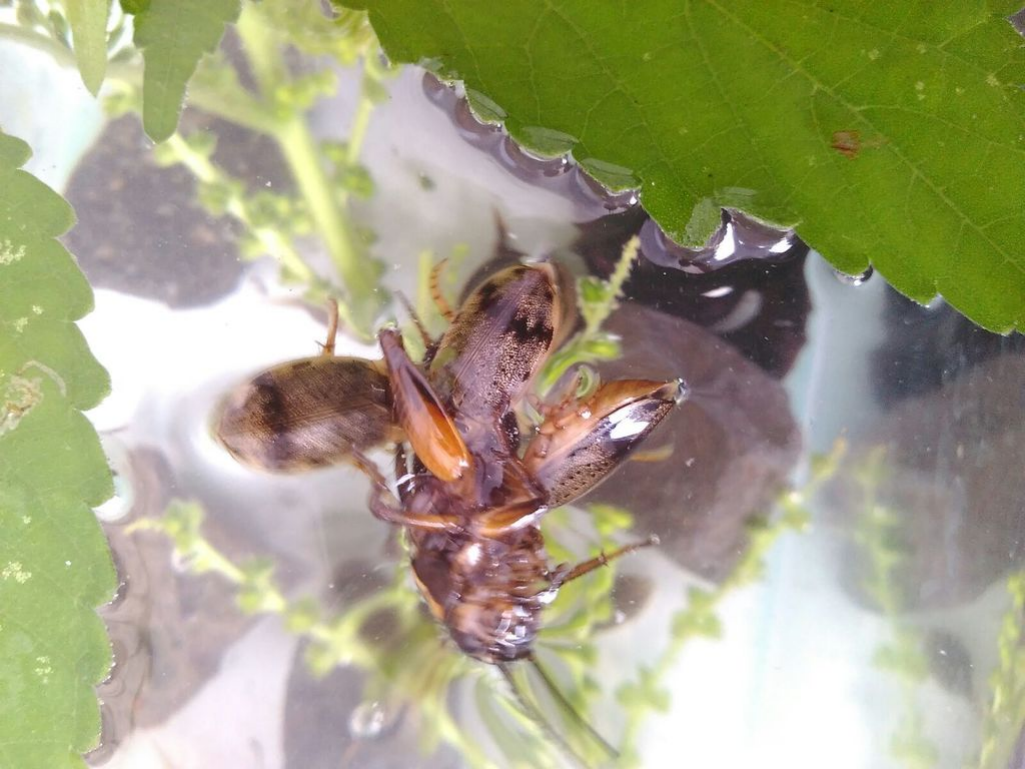
*Eretes griseus*, HP image from Bali.

**Figure 8.**
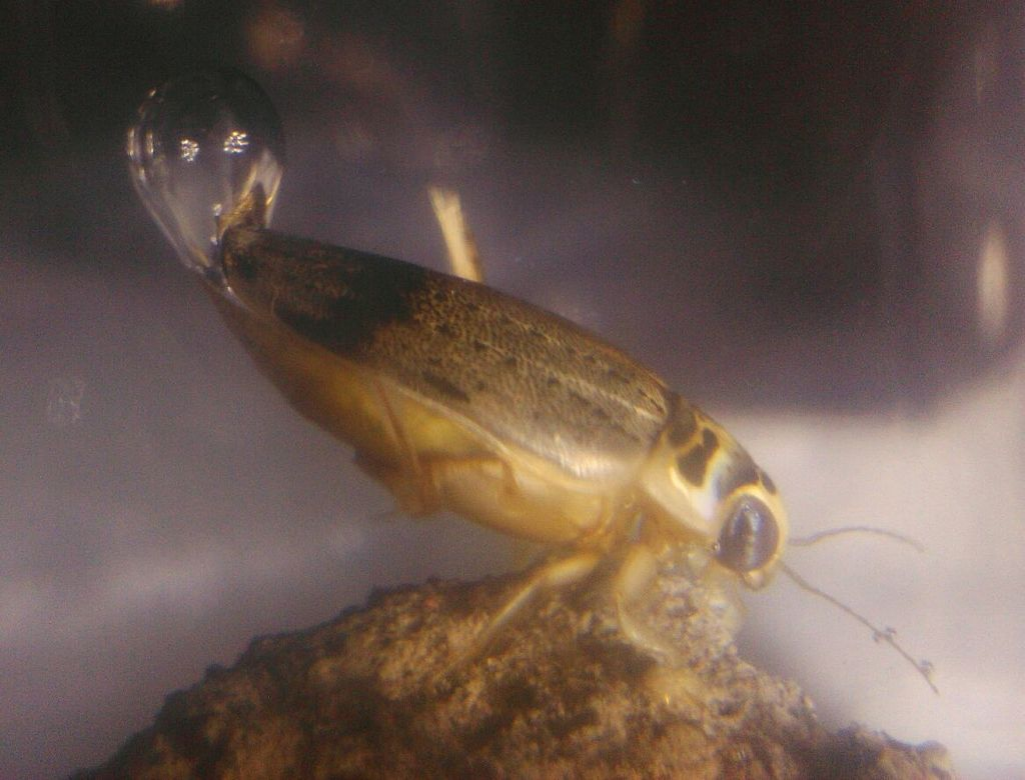
*Eretes griseus*, foto of Balinese specimen kept in aquarium.

*Eretes griseus* (*Figs 5, 6, 7*, Fig. 8).

*Hydaticus bipunctatus conjungens* (*Figs 3, 5*).

*Hydaticus fabricii* (*Fig. 3*).

**Figure 9.**
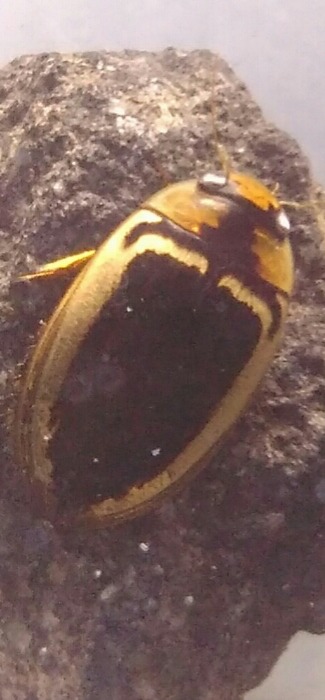
*Hydaticus luczonicus*, HP image from BALI_NS_2016_16.

*Hydaticus luczonicus* (*Fig. 9*).

**Figure 10.**
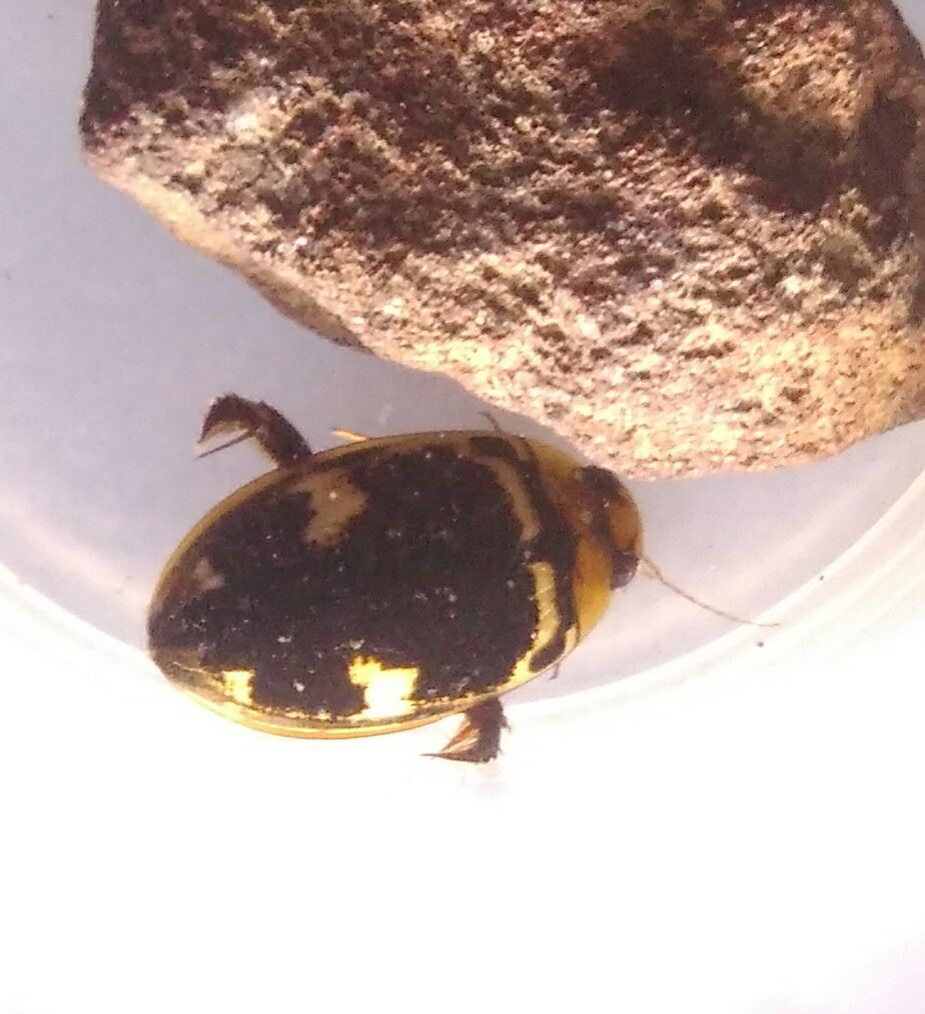
*Hydaticus pacificus*, HP image from BALI_NS_2016_17.

*Hydaticus pacificus* (*Fig. 10*).

**Figure 11.**
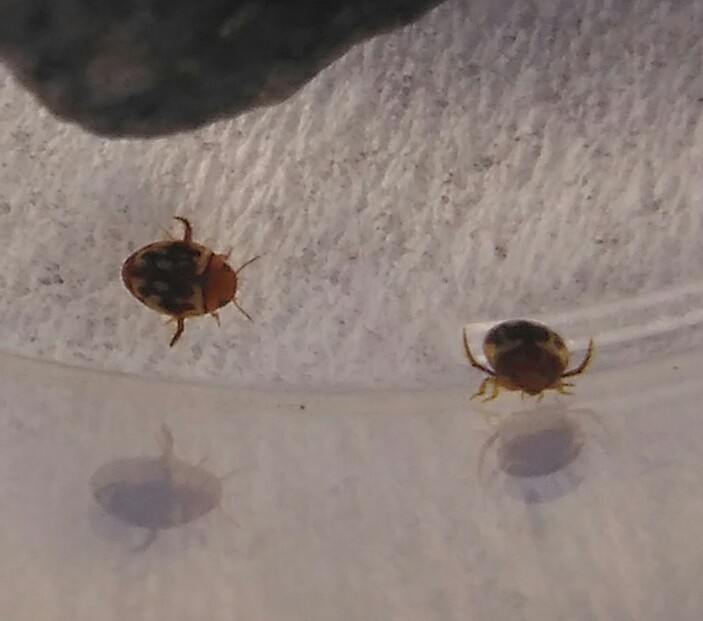
*Microdytes elgae*, HP image from BALI_NS_2016_07.

*Microdytes elgae* (*Fig. 11*).

**Figure 12.**
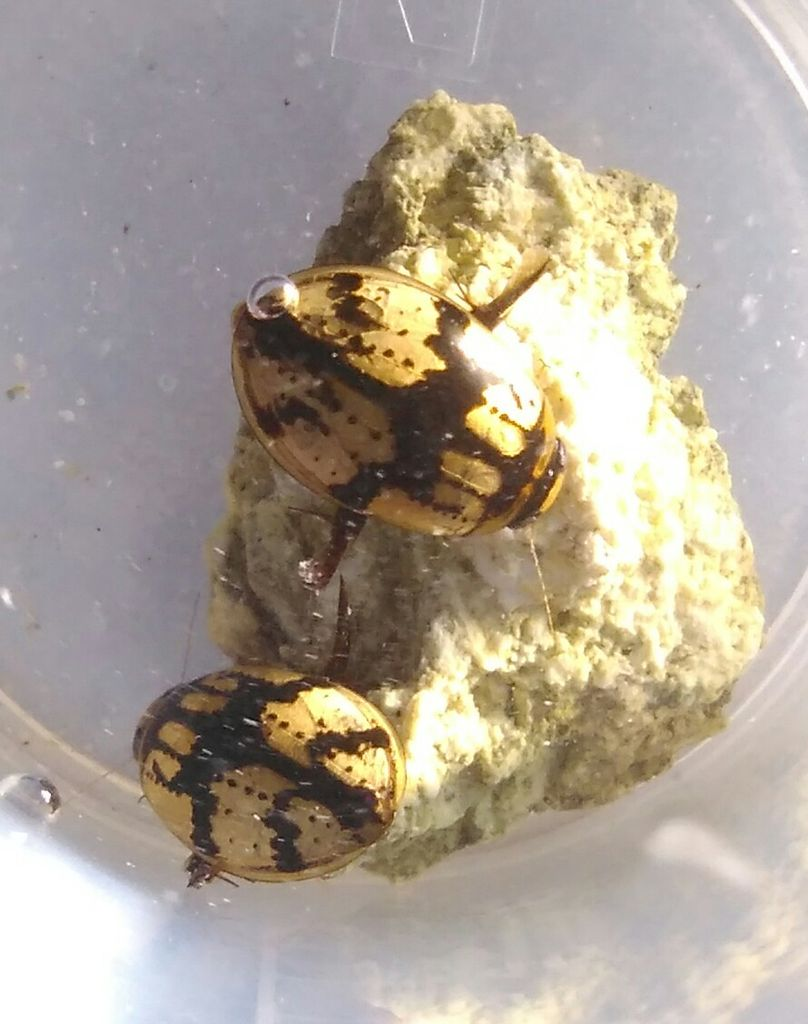
*Sandracottus hunteri*, HP image from BALI_NS_2016_18.

*Sandracottus hunteri* (*Fig. 12*).

## Taxon treatments

### Allopachria quadripustulata

Zimmermann, 1924

https://www.facebook.com/Baliwaterbeetles/posts/376240352500141

https://species-id.net/wiki/Allopachria_quadripustulata

Allopachria quadripustulata Zimmermann (1924): 195; Hendrich and Balke (1995): 41; Wewalka 2000: 101; Nilsson and Hájek 2017: 189.

#### Materials

- **Type status:** Other material. **Occurrence:** recordNumber: BALI_NS_2016_09; recordedBy: Suprayitno; individualCount: 1; lifeStage: adult; **Taxon:** scientificName: Allopachria quadripustulata; class: Insecta; order: Coleoptera; family: Dytiscidae; **Location:** island: Bali; country: Indonesia; stateProvince: Bali; county: Karangasem; locality: Manggis; verbatimElevation: 250m; locationRemarks: https://goo.gl/maps/rftZoZG1GGk; decimalLatitude: -8.484924; decimalLongitude: 115.528523; **Event:** samplingProtocol: collected with strainer; eventDate: 2016-09-10; **Record Level:** institutionCode: MZB; collectionCode: Entomology; ownerInstitutionCode: Museum Zoologicum Bogoriense
- **Type status:** Other material. **Occurrence:** recordNumber: BA 07 (1990); recordedBy: Hendrich and Balke; individualCount: 22; lifeStage: adult; **Taxon:** scientificName: Allopachria quadripustulata; class: Insecta; order: Coleoptera; family: Dytiscidae; **Location:** island: Bali; country: Indonesia; stateProvince: Bali; locality: Ubud, Monkey Forest; decimalLatitude: -8.517906; decimalLongitude: 115.258964; **Event:** samplingProtocol: collected with strainer; **Record Level:** institutionCode: ZSM etc; collectionCode: Entomology; ownerInstitutionCode: Zooligical State Collection Munich
- **Type status:** Other material. **Occurrence:** recordNumber: BALI_NS_2016_25; recordedBy: Suprayitno; individualCount: 1; lifeStage: adult; **Taxon:** scientificName: Allopachria quadripustulata; class: Insecta; order: Coleoptera; family: Dytiscidae; **Location:** island: Bali; country: Indonesia; stateProvince: Bali; county: Bangli; locality: Ds. Penida Kelod - Tembuku, Tukad Cepung Waterfall; verbatimElevation: 700m; locationRemarks: https://goo.gl/maps/5zAgEyGmuSp; decimalLatitude: -8.4401039; decimalLongitude: 115.3847355; **Event:** samplingProtocol: collected with strainer; eventDate: 2016-09-20; **Record Level:** institutionCode: MZB; collectionCode: Entomology; ownerInstitutionCode: Museum Zoologicum Bogoriense

#### Distribution in Bali

See Fig. 13.

#### Geographic range outside Bali

Indonesia: Sumatra, Siberut, Bali, Flores.

#### Ecology

**Figure 14.**
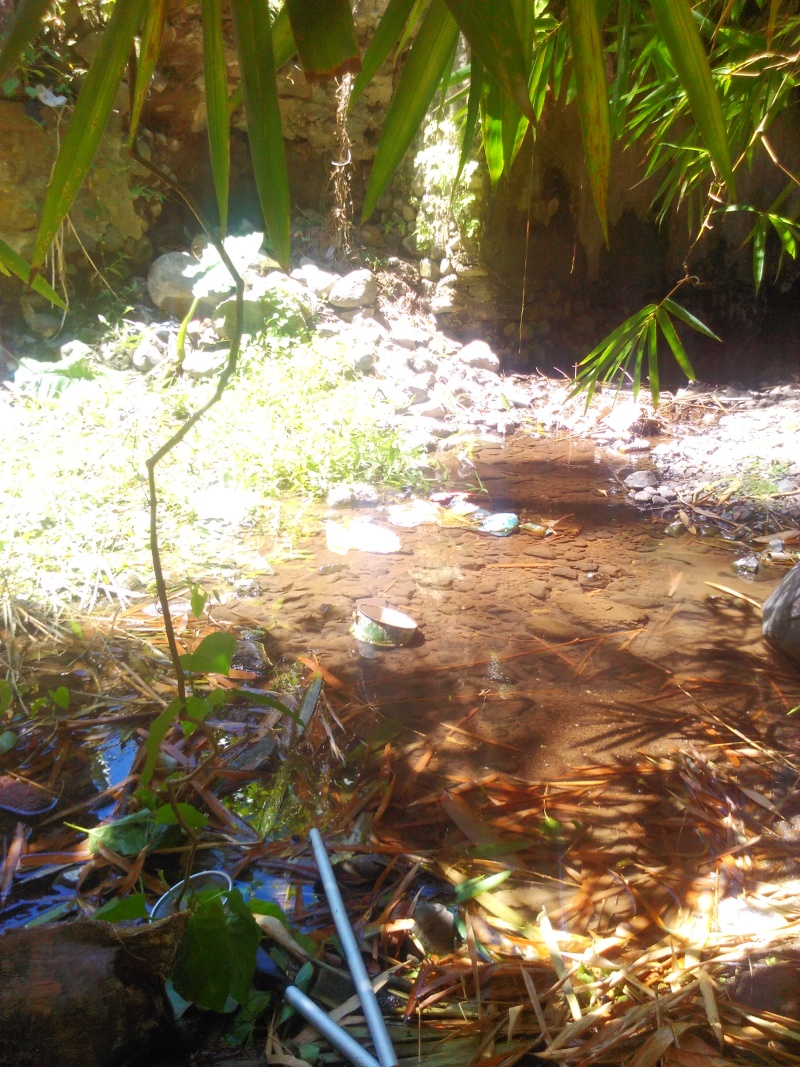
Collecting site BALI_NS_2016_09.

The species was collected from a shaded stream margin with volcanic gravel and roots, that site can be strongly flooded after rain (Hendrich & Balke 1995, Bali: Ubud, Monkey Forest, this site was revisited by Suprayitno several times but without beetle observations which might be due to heavy pollution of the stream). A similar habitat was now encountered in Manggis at locality BALI_NS_2016_09 (Fig. 14).

### Cybister tripunctatus temnenkii

Aubé, 1838

https://www.facebook.com/Baliwaterbeetles/photos/a.360137520777091.1073741831.359144747543035/368971266560383/?type=3&theater

Aubé (1838): 74; Hendrich and Balke 1995: 47; Nilsson and Hájek 2017: 77.

#### Materials

- **Type status:** Other material. **Occurrence:** recordNumber: BALI_NS_2016_05; recordedBy: Suprayitno; individualCount: 4; lifeStage: adult; **Taxon:** scientificName: Cybister tripunctatus; class: Insecta; order: Coleoptera; family: Dytiscidae; genus: Cybister; specificEpithet: tripunctatus temnenkii; scientificNameAuthorship: Aubé, 1838; **Location:** island: Bali; country: Indonesia; stateProvince: Bali; county: Karangasem; locality: Jl. Buana Giri - Duda Timur; verbatimElevation: 530m; locationRemarks: https://goo.gl/maps/mjErpSjYjLF2; decimalLatitude: -8.450549; decimalLongitude: 115.495006; **Event:** samplingProtocol: collected with strainer; eventDate: 08/30/2016; **Record Level:** institutionCode: MZB; collectionCode: Entomology; ownerInstitutionCode: Museum Zoologicum Bogoriense
- **Type status:** Other material. **Occurrence:** recordNumber: BALI_NS_2016_22; recordedBy: Suprayitno; individualCount: 1; lifeStage: adult; **Taxon:** scientificName: Cybister tripunctatus; class: Insecta; order: Coleoptera; family: Dytiscidae; genus: Cybister; specificEpithet: tripunctatus temnenkii; scientificNameAuthorship: Aubé, 1838; **Location:** island: Bali; country: Indonesia; stateProvince: Bali; county: Gianyar; locality: Batubulan; verbatimElevation: 30m; locationRemarks: http://maps.google.com/?q=-8.623022,115.267418&hl=en&gl=us; decimalLatitude: -8.623022; decimalLongitude: 115.267418; **Event:** samplingProtocol: collected with strainer; eventDate: 02/16/2016; **Record Level:** institutionCode: MZB; collectionCode: Entomology; ownerInstitutionCode: Museum Zoologicum Bogoriense
- **Type status:** Other material. **Occurrence:** recordedBy: Hermmann; individualCount: 1; lifeStage: adult; **Taxon:** scientificName: Cybister tripunctatus; class: Insecta; order: Coleoptera; family: Dytiscidae; genus: Cybister; specificEpithet: tripunctatus temnenkii; scientificNameAuthorship: Aubé, 1838; **Location:** island: Bali; country: Indonesia; stateProvince: Bali; county: Gianyar; locality: Tirta, Empul; verbatimElevation: 400m; decimalLatitude: -8.415371; decimalLongitude: 115.314854; **Event:** eventDate: 03/01/1986; **Record Level:** institutionCode: cAHerrmann; ownerInstitutionCode: Coll. A. Herrmann
- **Type status:** Other material. **Occurrence:** recordNumber: BALI_NS_2016_26; recordedBy: Suprayitno; individualCount: 1; lifeStage: adult; **Taxon:** scientificName: Cybister tripunctatus; class: Insecta; order: Coleoptera; family: Dytiscidae; genus: Cybister; specificEpithet: tripunctatus temnenkii; scientificNameAuthorship: Aubé, 1838; **Location:** island: Bali; country: Indonesia; stateProvince: Bali; county: Karangasem; locality: Jl. Amed, Desa Bunutan; verbatimElevation: 10m; locationRemarks: https://goo.gl/maps/as6BWjA2qmq; decimalLatitude: -8.346592; decimalLongitude: 115.669913; **Event:** samplingProtocol: collected with strainer; eventDate: 13.iv.2016 2016-04-13; **Record Level:** institutionCode: MZB; collectionCode: Entomology; ownerInstitutionCode: Museum Zoologicum Bogoriense
- **Type status:** Other material. **Occurrence:** recordNumber: BALI_NS_2016_33; recordedBy: Suprayitno; individualCount: 3; lifeStage: adult; **Taxon:** scientificName: Cybister tripunctatus; class: Insecta; order: Coleoptera; family: Dytiscidae; genus: Cybister; specificEpithet: tripunctatus temnenkii; scientificNameAuthorship: Aubé, 1838; **Location:** island: Bali; country: Indonesia; stateProvince: Bali; county: Denpasar; locality: Jl. Pantai Serangan, Serangan; verbatimElevation: 10m; locationRemarks: https://goo.gl/maps/5gZHFce9nGm; decimalLatitude: -8.732199; decimalLongitude: 115.235422; **Event:** samplingProtocol: collected with strainer; eventDate: 03/17/2016; **Record Level:** institutionCode: MZB; collectionCode: Entomology; ownerInstitutionCode: Museum Zoologicum Bogoriense
- **Type status:** Other material. **Occurrence:** recordNumber: BALI_NS_2016_34; recordedBy: Suprayitno; individualCount: 5; lifeStage: adult; **Taxon:** scientificName: Cybister tripunctatus; class: Insecta; order: Coleoptera; family: Dytiscidae; genus: Cybister; specificEpithet: tripunctatus temnenkii; scientificNameAuthorship: Aubé, 1838; **Location:** island: Bali; country: Indonesia; stateProvince: Bali; county: Badung; locality: Sedang village, Abiansemal; verbatimElevation: 120m; locationRemarks: http://maps.google.com/?q=-8.564203,115.237549&hl=en&gl=us; decimalLatitude: -8.564203; decimalLongitude: 115.237549; **Event:** samplingProtocol: collected with strainer; eventDate: 02/29/2016; **Record Level:** institutionCode: MZB; collectionCode: Entomology; ownerInstitutionCode: Museum Zoologicum Bogoriense
- **Type status:** Other material. **Occurrence:** recordNumber: BALI_NS_2016_48; recordedBy: Suprayitno; individualCount: 2; lifeStage: adult; **Taxon:** scientificName: Cybister tripunctatus; class: Insecta; order: Coleoptera; family: Dytiscidae; genus: Cybister; specificEpithet: tripunctatus temnenkii; scientificNameAuthorship: Aubé, 1838; **Location:** island: Bali; country: Indonesia; stateProvince: Bali; county: Klungkung; locality: Jl. Ped - Buyuk, Nusa Penida; verbatimElevation: 10m; locationRemarks: https://goo.gl/maps/zSg3MYd4y5E2; decimalLatitude: -8.677565; decimalLongitude: 115.52212; **Event:** samplingProtocol: collected with strainer; eventDate: 12/17/2016; **Record Level:** institutionCode: MZB; collectionCode: Entomology; ownerInstitutionCode: Museum Zoologicum Bogoriense
- **Type status:** Other material. **Occurrence:** recordedBy: Lars Hendrich observation; individualCount: >20; lifeStage: adult; **Taxon:** scientificName: Cybister tripunctatus; class: Insecta; order: Coleoptera; family: Dytiscidae; genus: Cybister; specificEpithet: tripunctatus temnenkii; scientificNameAuthorship: Aubé, 1838; **Location:** island: Bali; country: Indonesia; stateProvince: Bali; locality: Lake Beratan; verbatimElevation: 1200m; decimalLatitude: -8.281779; decimalLongitude: 115.165512; **Event:** samplingProtocol: observation at night
- **Type status:** Other material. **Occurrence:** recordedBy: Michael Balke & Ditta Amran; lifeStage: adult; **Taxon:** scientificName: Cybister tripunctatus; class: Insecta; order: Coleoptera; family: Dytiscidae; genus: Cybister; specificEpithet: tripunctatus temnenkii; scientificNameAuthorship: Aubé, 1838; **Location:** island: Bali; country: Indonesia; stateProvince: Bali; locality: Lake Tamblingan; decimalLatitude: -8.264136; decimalLongitude: 115.097851; **Event:** samplingProtocol: observation at day; eventDate: 2009

#### Distribution in Bali

See Fig. 15.

#### Geographic range outside Bali

The species is widespread in the Australian, Oriental and Palearctic regions; the nominal subspecies is Afrotropical.

#### Ecology

In a wide variety of stagnant water habitats such as lakes, ponds, paddies, where there is sufficient vegetation. Also in slowly streaming wide rivers with quieter, shallow and vegetated sections.

### Eretes griseus

(Fabricius, 1781)

https://www.facebook.com/Baliwaterbeetles/videos/552907661500075/

Eretes griseus Fabricius 1781: 293; Miller 2002: 262; Nilsson and Hájek 2017: 89.

#### Materials

- **Type status:** Other material. **Occurrence:** recordNumber: BALI_NS_2016_19; recordedBy: Suprayitno; individualCount: 1; lifeStage: adult; **Taxon:** scientificName: Eretes griseus; class: Insecta; order: Coleoptera; family: Dytiscidae; **Location:** island: Bali; country: Indonesia; stateProvince: Bali; county: Klungkung; locality: Jl. Raya Aan - Banjar Rangkan; verbatimElevation: 200m; locationRemarks: http://maps.google.com/?q=-8.514642,115.379928&hl=en&gl=us; decimalLatitude: -8.514642; decimalLongitude: 115.379928; **Event:** samplingProtocol: collected with strainer; eventDate: 2016-08-25; **Record Level:** institutionCode: MZB; collectionCode: Entomology; ownerInstitutionCode: Museum Zoologicum Bogoriense
- **Type status:** Other material. **Occurrence:** recordNumber: BALI_NS_2016_26; recordedBy: Suprayitno; individualCount: 1; lifeStage: adult; **Taxon:** scientificName: Eretes griseus; class: Insecta; order: Coleoptera; family: Dytiscidae; **Location:** island: Bali; country: Indonesia; stateProvince: Bali; county: Karangasem; locality: Jl. Amed, Desa Bunutan; verbatimElevation: 10m; locationRemarks: https://goo.gl/maps/as6BWjA2qmq; decimalLatitude: -8.346592; decimalLongitude: 115.669913; **Event:** samplingProtocol: collected with strainer; eventDate: 2016-04-13; **Record Level:** institutionCode: MZB; collectionCode: Entomology; ownerInstitutionCode: Museum Zoologicum Bogoriense
- **Type status:** Other material. **Occurrence:** recordNumber: BALI_NS_2016_33; recordedBy: Suprayitno; individualCount: 1; lifeStage: adult; **Taxon:** scientificName: Eretes griseus; class: Insecta; order: Coleoptera; family: Dytiscidae; **Location:** island: Bali; country: Indonesia; stateProvince: Bali; county: Denpasar; locality: Jl. Pantai Serangan, Serangan; verbatimElevation: 10m; locationRemarks: https://goo.gl/maps/5gZHFce9nGm; decimalLatitude: -8.732199; decimalLongitude: 115.235422; **Event:** samplingProtocol: collected with strainer; eventDate: 2016-03-07; **Record Level:** institutionCode: MZB; collectionCode: Entomology; ownerInstitutionCode: Museum Zoologicum Bogoriense
- **Type status:** Other material. **Occurrence:** recordNumber: BALI_NS_2016_37; recordedBy: Suprayitno; individualCount: 9; lifeStage: adult; **Taxon:** scientificName: Eretes griseus; class: Insecta; order: Coleoptera; family: Dytiscidae; **Location:** island: Bali; country: Indonesia; stateProvince: Bali; county: Klungkung; locality: Tukad Yeh Unda River; verbatimElevation: 6m; locationRemarks: https://goo.gl/maps/fxseqTb5b6N2; decimalLatitude: -8.561778; decimalLongitude: 115.43021; **Event:** samplingProtocol: collected with strainer; eventDate: 2016-02-05; **Record Level:** institutionCode: MZB; collectionCode: Entomology; ownerInstitutionCode: Museum Zoologicum Bogoriense
- **Type status:** Other material. **Occurrence:** recordNumber: BALI_NS_2016_34; recordedBy: Suprayitno; individualCount: 24; lifeStage: adult; **Taxon:** scientificName: Eretes griseus; class: Insecta; order: Coleoptera; family: Dytiscidae; **Location:** island: Bali; country: Indonesia; stateProvince: Bali; county: Badung; locality: Sedang village, Abiansemal; verbatimElevation: 120m; locationRemarks: http://maps.google.com/?q=-8.564203,115.237549&hl=en&gl=us; decimalLatitude: -8.564203; decimalLongitude: 115.237549; **Event:** samplingProtocol: collected with strainer; eventDate: 2016-02-29; **Record Level:** institutionCode: MZB; collectionCode: Entomology; ownerInstitutionCode: Museum Zoologicum Bogoriense
- **Type status:** Other material. **Occurrence:** recordNumber: BALI_NS_2016_48; recordedBy: Suprayitno; individualCount: 5; lifeStage: adult; **Taxon:** scientificName: Eretes griseus; class: Insecta; order: Coleoptera; family: Dytiscidae; **Location:** island: Bali; country: Indonesia; stateProvince: Bali; county: Klungkung; locality: Jl. Ped - Buyuk, Nusa Penida; verbatimElevation: 10m; locationRemarks: https://goo.gl/maps/zSg3MYd4y5E2; decimalLatitude: -8.677565; decimalLongitude: 115.52212; **Event:** samplingProtocol: collected with strainer; eventDate: 2016-12-17; **Record Level:** institutionCode: MZB; collectionCode: Entomology; ownerInstitutionCode: Museum Zoologicum Bogoriense
- **Type status:** Other material. **Occurrence:** recordNumber: BALI_NS_2017_59; recordedBy: Suprayitno; individualCount: 5; lifeStage: adult; **Taxon:** scientificName: Eretes griseus; class: Insecta; order: Coleoptera; family: Dytiscidae; **Location:** island: Bali; country: Indonesia; stateProvince: Bali; county: Karangasem; locality: Jl. Karangasem - Seraya; verbatimElevation: 10m; locationRemarks: https://goo.gl/maps/z4xE7VS3xfE2; decimalLatitude: -8.367711; decimalLongitude: 115.701407; **Event:** samplingProtocol: collected with strainer; eventDate: 2017-02-18; **Record Level:** institutionCode: MZB; collectionCode: Entomology; ownerInstitutionCode: Museum Zoologicum Bogoriense

#### Distribution in Bali

See Fig. 16.

#### Geographic range outside Bali

Widespread throughout warm regions of the Old World. First record for Bali.

#### Ecology

**Figure 17.**
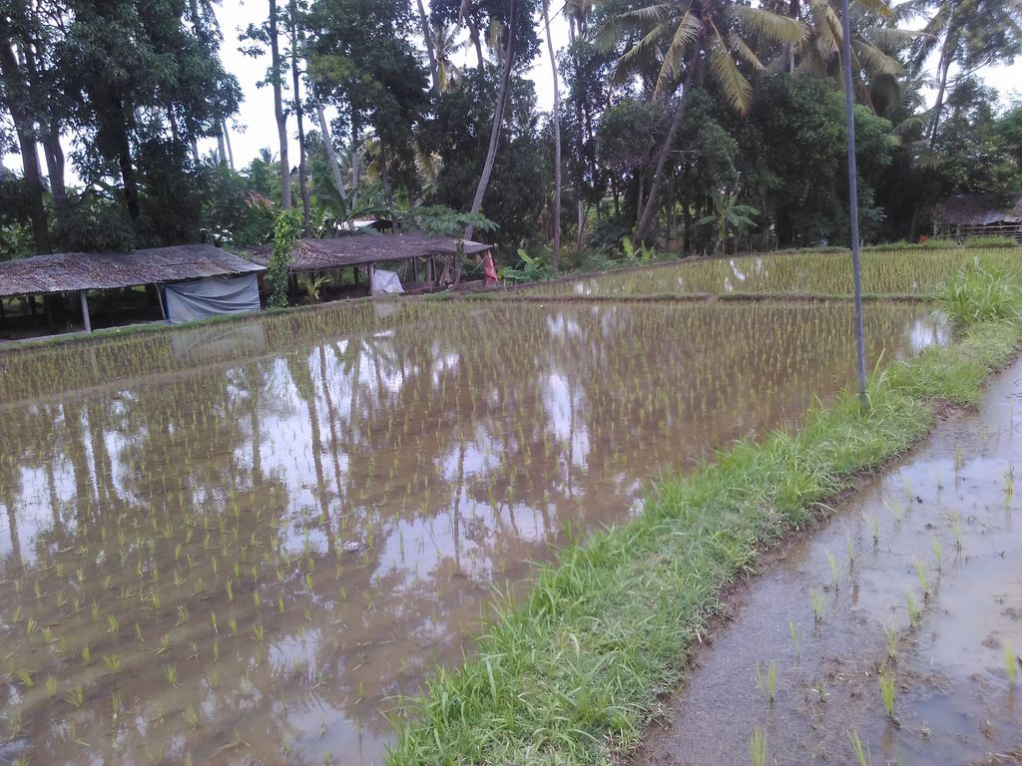
Collecting site BALI_NS_2016_34.

This seems to be a supertramp species, i.e. an early colonizer of stagnant, fully sun-exposed habitats on raw soil, with fine sand / clay. The beetles seem to dig into the substrate for cover. Often found at light (Fig. 17).

### Hydaticus bipunctatus conjungens

Régimbart, 1899

Régimbart 1899: 329; Hendrich and Balke 1995: 46; Nilsson and Hájek 2017: 91.

#### Materials

- **Type status:** Other material. **Occurrence:** recordNumber: BALI_NS_2016_04; recordedBy: Suprayitno; individualCount: 2; lifeStage: adult; **Taxon:** scientificName: Hydaticus bipuncatatus conjungens; class: Insecta; order: Coleoptera; family: Dytiscidae; genus: Hydaticus; specificEpithet: Hydaticus bipuncatatus conjungens; scientificNameAuthorship: Régimbart, 1899; **Location:** island: Bali; country: Indonesia; stateProvince: Bali; county: Karangasem; locality: Jl. Gegelang - Duda Timur; verbatimElevation: 460m; locationRemarks: https://goo.gl/maps/ti5uRJuMQYu; decimalLatitude: -8.469917; decimalLongitude: 115.487944; **Event:** samplingProtocol: collected with strainer; eventDate: 08/30/2016; **Record Level:** institutionCode: MZB; collectionCode: Entomology; ownerInstitutionCode: Museum Zoologicum Bogoriense
- **Type status:** Other material. **Occurrence:** recordNumber: BALI_NS_2016_05; recordedBy: Suprayitno; individualCount: 4; lifeStage: adult; **Taxon:** scientificName: Hydaticus bipuncatatus conjungens; class: Insecta; order: Coleoptera; family: Dytiscidae; genus: Hydaticus; specificEpithet: Hydaticus bipuncatatus conjungens; scientificNameAuthorship: Régimbart, 1899; **Location:** island: Bali; country: Indonesia; stateProvince: Bali; county: Karangasem; locality: Jl. Buana Giri - Duda Timur; verbatimElevation: 530m; locationRemarks: https://goo.gl/maps/mjErpSjYjLF2; decimalLatitude: -8.450549; decimalLongitude: 115.495006; **Event:** samplingProtocol: collected with strainer; eventDate: 08/30/2016; **Record Level:** institutionCode: MZB; collectionCode: Entomology; ownerInstitutionCode: Museum Zoologicum Bogoriense
- **Type status:** Other material. **Occurrence:** recordNumber: BALI_NS_2016_06; recordedBy: Suprayitno; individualCount: 6; lifeStage: adult; **Taxon:** scientificName: Hydaticus bipuncatatus conjungens; class: Insecta; order: Coleoptera; family: Dytiscidae; genus: Hydaticus; specificEpithet: Hydaticus bipuncatatus conjungens; scientificNameAuthorship: Régimbart, 1899; **Location:** island: Bali; country: Indonesia; stateProvince: Bali; county: Karangasem; locality: Jl. Pura Pucak Kaler; verbatimElevation: 60m; locationRemarks: https://goo.gl/maps/LsgLwZ8kgPk; decimalLatitude: -8.501913; decimalLongitude: 115.483401; **Event:** samplingProtocol: collected with strainer; eventDate: 09/01/2016; **Record Level:** institutionCode: MZB; collectionCode: Entomology; ownerInstitutionCode: Museum Zoologicum Bogoriense
- **Type status:** Other material. **Occurrence:** recordNumber: BALI_NS_2016_12; recordedBy: Suprayitno; individualCount: 2; lifeStage: adult; **Taxon:** scientificName: Hydaticus bipuncatatus conjungens; class: Insecta; order: Coleoptera; family: Dytiscidae; genus: Hydaticus; specificEpithet: Hydaticus bipuncatatus conjungens; scientificNameAuthorship: Régimbart, 1899; **Location:** island: Bali; country: Indonesia; stateProvince: Bali; county: Tabanan; locality: Baturiti; verbatimElevation: 830m; locationRemarks: https://goo.gl/maps/WWGpxwqfgFm; decimalLatitude: -8.325723; decimalLongitude: 115.195042; **Event:** samplingProtocol: collected with strainer; eventDate: 09/24/2016; **Record Level:** institutionCode: MZB; collectionCode: Entomology; ownerInstitutionCode: Museum Zoologicum Bogoriense
- **Type status:** Other material. **Occurrence:** recordNumber: BALI_NS_2016_18; recordedBy: Suprayitno; individualCount: 1; lifeStage: adult; **Taxon:** scientificName: Hydaticus bipuncatatus conjungens; class: Insecta; order: Coleoptera; family: Dytiscidae; genus: Hydaticus; specificEpithet: Hydaticus bipuncatatus conjungens; scientificNameAuthorship: Régimbart, 1899; **Location:** island: Bali; country: Indonesia; stateProvince: Bali; county: Karangasem; locality: Jalan Karangasem - Seraya (2); verbatimElevation: 270m; locationRemarks: https://goo.gl/maps/MRMj83F9AAz; decimalLatitude: -8.421717; decimalLongitude: 115.669085; **Event:** samplingProtocol: collected with strainer; eventDate: 06/04/2016; **Record Level:** institutionCode: MZB; collectionCode: Entomology; ownerInstitutionCode: Museum Zoologicum Bogoriense
- **Type status:** Other material. **Occurrence:** recordNumber: BALI_NS_2016_19; recordedBy: Suprayitno; individualCount: 3; lifeStage: adult; **Taxon:** scientificName: Hydaticus bipuncatatus conjungens; class: Insecta; order: Coleoptera; family: Dytiscidae; genus: Hydaticus; specificEpithet: Hydaticus bipuncatatus conjungens; scientificNameAuthorship: Régimbart, 1899; **Location:** island: Bali; country: Indonesia; stateProvince: Bali; county: Karangasem; locality: Jalan Karangasem - Seraya (3); verbatimElevation: 270m; locationRemarks: https://goo.gl/maps/GJpzEosySy12; decimalLatitude: -8.421574; decimalLongitude: 115.671857; **Event:** samplingProtocol: collected with strainer; eventDate: 06/05/2016; **Record Level:** institutionCode: MZB; collectionCode: Entomology; ownerInstitutionCode: Museum Zoologicum Bogoriense
- **Type status:** Other material. **Occurrence:** recordNumber: BALI_NS_2016_20; recordedBy: Suprayitno; individualCount: 5; lifeStage: adult; **Taxon:** scientificName: Hydaticus bipuncatatus conjungens; class: Insecta; order: Coleoptera; family: Dytiscidae; genus: Hydaticus; specificEpithet: Hydaticus bipuncatatus conjungens; scientificNameAuthorship: Régimbart, 1899; **Location:** island: Bali; country: Indonesia; stateProvince: Bali; county: Karangasem; locality: Jalan Karangasem - Seraya (5); verbatimElevation: 170m; locationRemarks: https://goo.gl/maps/fAT8hjLK4Y82; decimalLatitude: -8.417738; decimalLongitude: 115.688551; **Event:** samplingProtocol: collected with strainer; eventDate: 06/16/2016; **Record Level:** institutionCode: MZB; collectionCode: Entomology; ownerInstitutionCode: Museum Zoologicum Bogoriense
- **Type status:** Other material. **Occurrence:** recordNumber: BALI_NS_2016_23; recordedBy: Suprayitno; individualCount: 2; lifeStage: adult; **Taxon:** scientificName: Hydaticus bipuncatatus conjungens; class: Insecta; order: Coleoptera; family: Dytiscidae; genus: Hydaticus; specificEpithet: Hydaticus bipuncatatus conjungens; scientificNameAuthorship: Régimbart, 1899; **Location:** island: Bali; country: Indonesia; stateProvince: Bali; county: Badung; locality: Desa Ungasan near Pecatu; verbatimElevation: 140m; locationRemarks: https://goo.gl/maps/p64cgybQRDF2; decimalLatitude: -8.840126; decimalLongitude: 115.144003; **Event:** samplingProtocol: collected with strainer; eventDate: 06/20/2016; **Record Level:** institutionCode: MZB; collectionCode: Entomology; ownerInstitutionCode: Museum Zoologicum Bogoriense
- **Type status:** Other material. **Occurrence:** recordNumber: BALI_NS_2016_26; recordedBy: Suprayitno; individualCount: 1; lifeStage: adult; **Taxon:** scientificName: Hydaticus bipuncatatus conjungens; class: Insecta; order: Coleoptera; family: Dytiscidae; genus: Hydaticus; specificEpithet: Hydaticus bipuncatatus conjungens; scientificNameAuthorship: Régimbart, 1899; **Location:** island: Bali; country: Indonesia; stateProvince: Bali; county: Karangasem; locality: Jl. Amed, Desa Bunutan; verbatimElevation: 10m; locationRemarks: https://goo.gl/maps/as6BWjA2qmq; decimalLatitude: -8.346592; decimalLongitude: 115.669913; **Event:** samplingProtocol: collected with strainer; eventDate: 04/13/2016; **Record Level:** institutionCode: MZB; collectionCode: Entomology; ownerInstitutionCode: Museum Zoologicum Bogoriense
- **Type status:** Other material. **Occurrence:** recordNumber: BALI_NS_2016_31; recordedBy: Suprayitno; individualCount: 3; lifeStage: adult; **Taxon:** scientificName: Hydaticus bipuncatatus conjungens; class: Insecta; order: Coleoptera; family: Dytiscidae; genus: Hydaticus; specificEpithet: Hydaticus bipuncatatus conjungens; scientificNameAuthorship: Régimbart, 1899; **Location:** island: Bali; country: Indonesia; stateProvince: Bali; county: Gianyar; locality: Jl. Katiklantang, Ds. Singakerta, Ubud; verbatimElevation: 190m; locationRemarks: https://goo.gl/maps/tcZcCJwQrzT2; decimalLatitude: -8.517321; decimalLongitude: 115.251018; **Event:** samplingProtocol: collected with strainer; eventDate: 07/06/2016; **Record Level:** institutionCode: MZB; collectionCode: Entomology; ownerInstitutionCode: Museum Zoologicum Bogoriense
- **Type status:** Other material. **Occurrence:** recordNumber: BALI_NS_2016_33; recordedBy: Suprayitno; individualCount: 1; lifeStage: adult; **Taxon:** scientificName: Hydaticus bipuncatatus conjungens; class: Insecta; order: Coleoptera; family: Dytiscidae; genus: Hydaticus; specificEpithet: Hydaticus bipuncatatus conjungens; scientificNameAuthorship: Régimbart, 1899; **Location:** island: Bali; country: Indonesia; stateProvince: Bali; county: Denpasar; locality: Jl. Pantai Serangan, Serangan; verbatimElevation: 10m; locationRemarks: https://goo.gl/maps/5gZHFce9nGm; decimalLatitude: -8.732199; decimalLongitude: 115.235422; **Event:** samplingProtocol: collected with strainer; eventDate: 03/07/2016; **Record Level:** institutionCode: MZB; collectionCode: Entomology; ownerInstitutionCode: Museum Zoologicum Bogoriense
- **Type status:** Other material. **Occurrence:** recordNumber: BALI_NS_2016_39; recordedBy: Suprayitno; individualCount: 5; lifeStage: adult; **Taxon:** scientificName: Hydaticus bipuncatatus conjungens; class: Insecta; order: Coleoptera; family: Dytiscidae; genus: Hydaticus; specificEpithet: Hydaticus bipuncatatus conjungens; scientificNameAuthorship: Régimbart, 1899; **Location:** island: Bali; country: Indonesia; stateProvince: Bali; county: Karangasem; locality: Jl. Raya Yehpoh - Manggis; verbatimElevation: 50m; locationRemarks: http://maps.google.com/?q=-8.486125,115.516439&hl=en&gl=us; decimalLatitude: -8.486125; decimalLongitude: 115.516439; **Event:** samplingProtocol: collected with strainer; eventDate: 11/05/2016; **Record Level:** institutionCode: MZB; collectionCode: Entomology; ownerInstitutionCode: Museum Zoologicum Bogoriense
- **Type status:** Other material. **Occurrence:** recordNumber: BALI_NS_2016_51; recordedBy: Suprayitno; individualCount: 1; lifeStage: adult; **Taxon:** scientificName: Hydaticus bipuncatatus conjungens; class: Insecta; order: Coleoptera; family: Dytiscidae; genus: Hydaticus; specificEpithet: Hydaticus bipuncatatus conjungens; scientificNameAuthorship: Régimbart, 1899; **Location:** island: Bali; country: Indonesia; stateProvince: Bali; county: Gianyar; locality: Jl. Yudistira - Batubulan; verbatimElevation: 40m; locationRemarks: https://goo.gl/maps/sQHVTyGEDkA2; decimalLatitude: -8.624977; decimalLongitude: 115.276241; **Event:** samplingProtocol: collected with strainer; eventDate: 12/31/2016; **Record Level:** institutionCode: MZB; collectionCode: Entomology; ownerInstitutionCode: Museum Zoologicum Bogoriense
- **Type status:** Other material. **Occurrence:** recordNumber: BALI_NS_2017_54; recordedBy: Suprayitno; individualCount: 1; lifeStage: adult; **Taxon:** scientificName: Hydaticus bipuncatatus conjungens; class: Insecta; order: Coleoptera; family: Dytiscidae; genus: Hydaticus; specificEpithet: Hydaticus bipuncatatus conjungens; scientificNameAuthorship: Régimbart, 1899; **Location:** island: Bali; country: Indonesia; stateProvince: Bali; county: Bangli; locality: Central Batur - Kintamani; verbatimElevation: 1000m; locationRemarks: https://goo.gl/maps/btGBsiLJjxm; decimalLatitude: -8.266562; decimalLongitude: 115.381414; **Event:** samplingProtocol: collected with strainer; eventDate: 01/08/2017; **Record Level:** institutionCode: MZB; collectionCode: Entomology; ownerInstitutionCode: Museum Zoologicum Bogoriense
- **Type status:** Other material. **Occurrence:** recordNumber: BALI_NS_2017_59; recordedBy: Suprayitno; individualCount: 3; lifeStage: adult; **Taxon:** scientificName: Hydaticus bipuncatatus conjungens; class: Insecta; order: Coleoptera; family: Dytiscidae; genus: Hydaticus; specificEpithet: Hydaticus bipuncatatus conjungens; scientificNameAuthorship: Régimbart, 1899; **Location:** island: Bali; country: Indonesia; stateProvince: Bali; county: Karangasem; locality: Jl. Karangasem - Seraya; verbatimElevation: 10m; locationRemarks: https://goo.gl/maps/z4xE7VS3xfE2; decimalLatitude: -8.367711; decimalLongitude: 115.701407; **Event:** samplingProtocol: collected with strainer; eventDate: 02/18/2017; **Record Level:** institutionCode: MZB; collectionCode: Entomology; ownerInstitutionCode: Museum Zoologicum Bogoriense
- **Type status:** Other material. **Occurrence:** recordNumber: BALI_NS_2017_60; recordedBy: Suprayitno; individualCount: 1; lifeStage: adult; **Taxon:** scientificName: Hydaticus bipuncatatus conjungens; class: Insecta; order: Coleoptera; family: Dytiscidae; genus: Hydaticus; specificEpithet: Hydaticus bipuncatatus conjungens; scientificNameAuthorship: Régimbart, 1899; **Location:** island: Bali; country: Indonesia; stateProvince: Bali; county: Karangasem; locality: Unname road - Bunutan - Abang; verbatimElevation: 100m; locationRemarks: https://goo.gl/maps/SPcH1zJWEVD2; decimalLatitude: -8.359665; decimalLongitude: 115.66442; **Event:** samplingProtocol: collected with strainer; eventDate: 02/19/2017; **Record Level:** institutionCode: MZB; collectionCode: Entomology; ownerInstitutionCode: Museum Zoologicum Bogoriense
- **Type status:** Other material. **Occurrence:** recordNumber: BALI_NS_2016_61; recordedBy: Suprayitno; individualCount: 5; lifeStage: adult; **Taxon:** scientificName: Hydaticus bipuncatatus conjungens; class: Insecta; order: Coleoptera; family: Dytiscidae; genus: Hydaticus; specificEpithet: Hydaticus bipuncatatus conjungens; scientificNameAuthorship: Régimbart, 1899; **Location:** island: Bali; country: Indonesia; stateProvince: Bali; county: Karangasem; locality: Unnamed road - Bunutan - Abang; verbatimElevation: 100m; locationRemarks: https://goo.gl/maps/Efa9yguQBB32; decimalLatitude: -8.360504; decimalLongitude: 115.662144; **Event:** samplingProtocol: collected with strainer; eventDate: 04/14/2016; **Record Level:** institutionCode: MZB; collectionCode: Entomology; ownerInstitutionCode: Museum Zoologicum Bogoriense
- **Type status:** Other material. **Occurrence:** recordNumber: BALI_NS_2017_62; recordedBy: Suprayitno; individualCount: 1; lifeStage: adult; **Taxon:** scientificName: Hydaticus bipuncatatus conjungens; class: Insecta; order: Coleoptera; family: Dytiscidae; genus: Hydaticus; specificEpithet: Hydaticus bipuncatatus conjungens; scientificNameAuthorship: Régimbart, 1899; **Location:** island: Bali; country: Indonesia; stateProvince: Bali; county: Klungkung; locality: Desa Selat, Klungkung, Alam rafting finish point river; verbatimElevation: 240m; locationRemarks: http://maps.google.com/?q=-8.478910,115.410956&hl=en&gl=us; decimalLatitude: -8.47891; decimalLongitude: 115.410956; **Event:** samplingProtocol: collected with strainer; eventDate: 04/13/2017; **Record Level:** institutionCode: MZB; collectionCode: Entomology; ownerInstitutionCode: Museum Zoologicum Bogoriense
- **Type status:** Other material. **Occurrence:** recordNumber: BALI_NS_2017_63; recordedBy: Suprayitno; individualCount: 2; lifeStage: adult; **Taxon:** scientificName: Hydaticus bipuncatatus conjungens; class: Insecta; order: Coleoptera; family: Dytiscidae; genus: Hydaticus; specificEpithet: Hydaticus bipuncatatus conjungens; scientificNameAuthorship: Régimbart, 1899; **Location:** island: Bali; country: Indonesia; stateProvince: Bali; county: Karangasem; locality: Jl. Melasti, Purwakerti, Abang; verbatimElevation: 50m; locationRemarks: https://goo.gl/maps/MaPhfyqF8Du; decimalLatitude: -8.330304; decimalLongitude: 115.637501; **Event:** samplingProtocol: collected with strainer; eventDate: 04/11/2017; **Record Level:** institutionCode: MZB; collectionCode: Entomology; ownerInstitutionCode: Museum Zoologicum Bogoriense
- **Type status:** Other material. **Occurrence:** recordNumber: BA93-2; recordedBy: Balke; individualCount: 1; lifeStage: adult; **Taxon:** scientificName: Hydaticus bipuncatatus conjungens; class: Insecta; order: Coleoptera; family: Dytiscidae; genus: Hydaticus; specificEpithet: Hydaticus bipuncatatus conjungens; scientificNameAuthorship: Régimbart, 1899; **Location:** island: Bali; country: Indonesia; stateProvince: Bali; county: Gianyar; locality: Ubud; verbatimElevation: 180m; decimalLatitude: -8.516324; decimalLongitude: 115.257336; **Event:** samplingProtocol: collected with strainer; eventDate: 10/27/1993; **Record Level:** institutionCode: ZSM; collectionCode: Entomology; ownerInstitutionCode: Zooligical State Collection Munich
- **Type status:** Other material. **Occurrence:** recordNumber: BA9; recordedBy: Hendrich & Balke; individualCount: 40; lifeStage: adult; **Taxon:** scientificName: Hydaticus bipuncatatus conjungens; class: Insecta; order: Coleoptera; family: Dytiscidae; genus: Hydaticus; specificEpithet: Hydaticus bipuncatatus conjungens; scientificNameAuthorship: Régimbart, 1899; **Location:** island: Bali; country: Indonesia; stateProvince: Bali; county: Buleleng; locality: Lake Buyan; verbatimElevation: 1250m; decimalLatitude: -8.251232; decimalLongitude: 115.129773; **Event:** samplingProtocol: dipnet; eventDate: 07/12/1991; **Record Level:** institutionCode: ZSM; collectionCode: Entomology; ownerInstitutionCode: Zooligical State Collection Munich

#### Distribution in Bali

See Fig. 18.

#### Geographic range outside Bali

Sunda islands as far east as Sumba, Sulawesi.

#### Ecology

**Figure 19.**
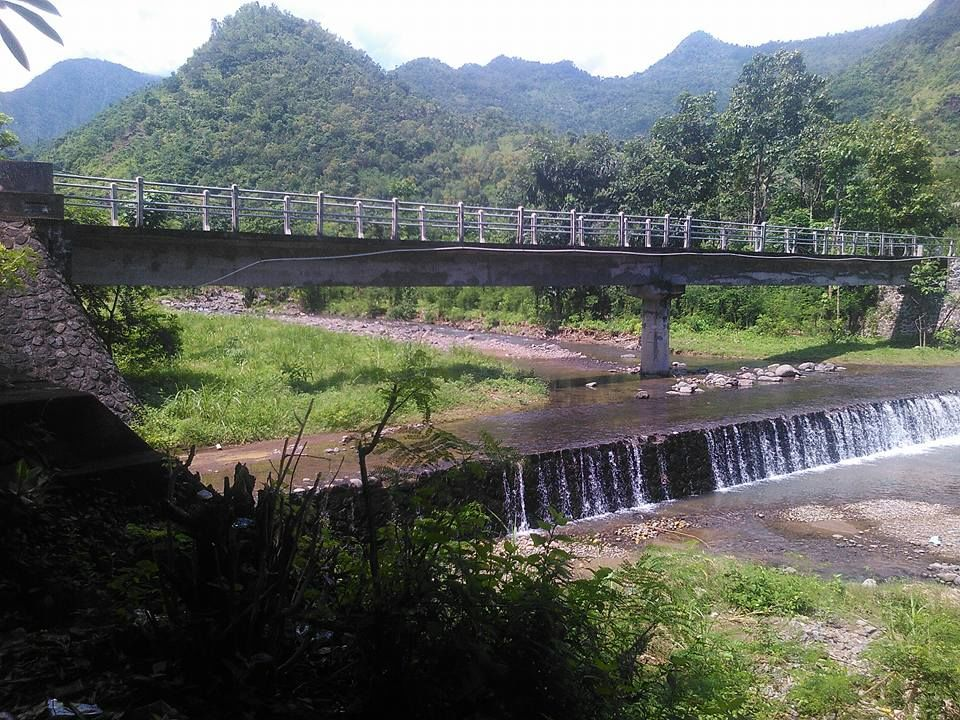
Collecting site BALI_NS_2016_60.

**Figure 20.**
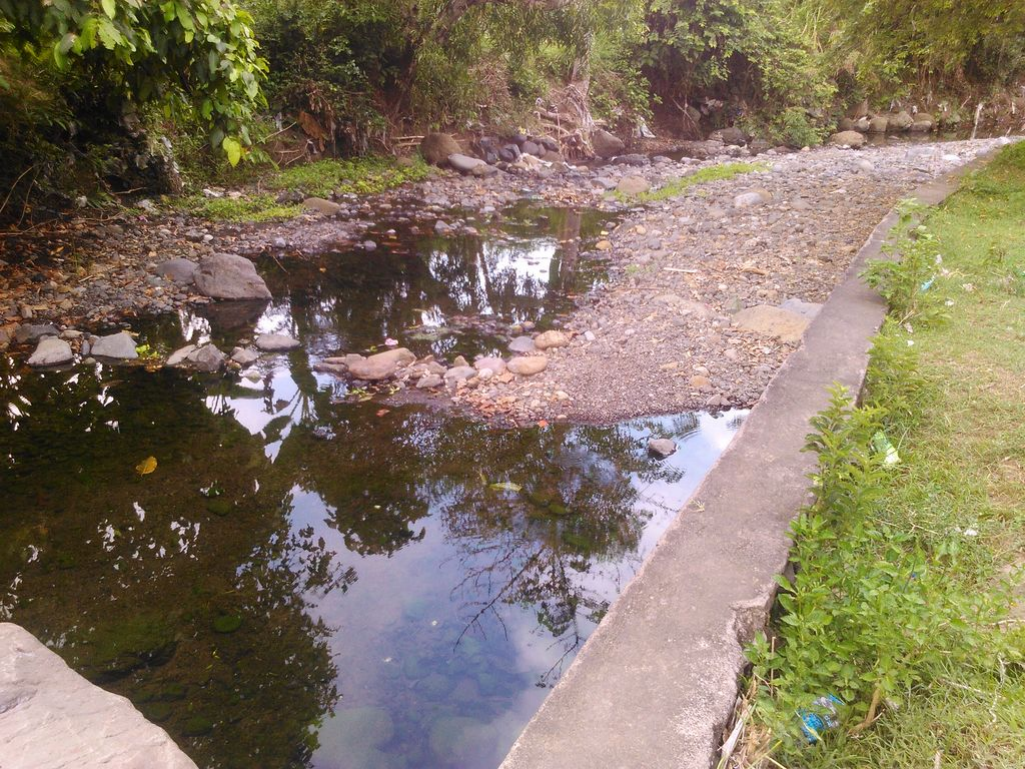
Collecting site BALI_NS_2016_39.

The species is occupies stagnant water habitats with emergent vegetation or pools on rocky / gravelly ground at the edge of streams and creeks, sometimes also roadside pools (Figs 19, 20).

### Hydaticus fabricii

(W.S. Macleay, 1825)

https://www.facebook.com/Baliwaterbeetles/photos/a.360137520777091.1073741831.359144747543035/442314512559391/?type=3&theater

Hydaticus fabricii Wewalka 1979: 121;Hendrich and Balke 1995: 46; Nilsson and Hájek 2017: 92.

#### Materials

- **Type status:** Other material. **Occurrence:** recordNumber: BALI_NS_2016_04; recordedBy: Suprayitno; individualCount: 2; lifeStage: adult; **Taxon:** scientificName: Hydaticus fabricii; class: Insecta; order: Coleoptera; family: Dytiscidae; **Location:** island: Bali; country: Indonesia; stateProvince: Bali; county: Karangasem; locality: Jl. Gegelang - Duda Timur; verbatimElevation: 460m; locationRemarks: https://goo.gl/maps/ti5uRJuMQYu; decimalLatitude: -8.469917; decimalLongitude: 115.487944; **Event:** samplingProtocol: collected with strainer; samplingEffort: 2016-08-30; eventDate: 30.viii.2016; **Record Level:** institutionCode: MZB; collectionCode: Entomology; ownerInstitutionCode: Museum Zoologicum Bogoriense
- **Type status:** Other material. **Occurrence:** recordNumber: BALI_NS_2016_05; recordedBy: Suprayitno; individualCount: 1; lifeStage: adult; **Taxon:** scientificName: Hydaticus fabricii; class: Insecta; order: Coleoptera; family: Dytiscidae; **Location:** island: Bali; country: Indonesia; stateProvince: Bali; county: Karangasem; locality: Jl. Buana Giri - Duda Timur; verbatimElevation: 530m; locationRemarks: https://goo.gl/maps/mjErpSjYjLF2; decimalLatitude: -8.450549; decimalLongitude: 115.495006; **Event:** samplingProtocol: collected with strainer; samplingEffort: 2016-08-30; eventDate: 30.viii.2016; **Record Level:** institutionCode: MZB; collectionCode: Entomology; ownerInstitutionCode: Museum Zoologicum Bogoriense
- **Type status:** Other material. **Occurrence:** recordNumber: BALI_NS_2016_06; recordedBy: Suprayitno; individualCount: 1; lifeStage: adult; **Taxon:** scientificName: Hydaticus fabricii; class: Insecta; order: Coleoptera; family: Dytiscidae; **Location:** island: Bali; country: Indonesia; stateProvince: Bali; county: Karangasem; locality: Jl. Pura Pucak Kaler; verbatimElevation: 60m; locationRemarks: https://goo.gl/maps/LsgLwZ8kgPk; decimalLatitude: -8.501913; decimalLongitude: 115.483401; **Event:** samplingProtocol: collected with strainer; samplingEffort: 2016-09-01; eventDate: 1.ix.2016; **Record Level:** institutionCode: MZB; collectionCode: Entomology; ownerInstitutionCode: Museum Zoologicum Bogoriense
- **Type status:** Other material. **Occurrence:** recordNumber: BALI_NS_2016_12; recordedBy: Suprayitno; individualCount: 1; lifeStage: adult; **Taxon:** scientificName: Hydaticus fabricii; class: Insecta; order: Coleoptera; family: Dytiscidae; **Location:** island: Bali; country: Indonesia; stateProvince: Bali; county: Tabanan; locality: Baturiti; verbatimElevation: 830m; locationRemarks: https://goo.gl/maps/WWGpxwqfgFm; decimalLatitude: -8.325723; decimalLongitude: 115.195042; **Event:** samplingProtocol: collected with strainer; samplingEffort: 2016-09-24; eventDate: 24.ix.2016; **Record Level:** institutionCode: MZB; collectionCode: Entomology; ownerInstitutionCode: Museum Zoologicum Bogoriense
- **Type status:** Other material. **Occurrence:** recordNumber: BALI_NS_2016_01; recordedBy: Suprayitno; individualCount: 1; lifeStage: adult; **Taxon:** scientificName: Hydaticus fabricii; class: Insecta; order: Coleoptera; family: Dytiscidae; **Location:** island: Bali; country: Indonesia; stateProvince: Bali; county: Klungkung; locality: Jl. Raya Aan - Banjar Rangkan; verbatimElevation: 200m; locationRemarks: http://maps.google.com/?q=-8.514642,115.379928&hl=en&gl=us; decimalLatitude: -8.514642; decimalLongitude: 115.379928; **Event:** samplingProtocol: collected with strainer; samplingEffort: 2016-08-25; eventDate: 25.viii.2016; **Record Level:** institutionCode: MZB; collectionCode: Entomology; ownerInstitutionCode: Museum Zoologicum Bogoriense
- **Type status:** Other material. **Occurrence:** recordNumber: BALI_NS_2016_09; recordedBy: Suprayitno; individualCount: 1; lifeStage: adult; **Taxon:** scientificName: Hydaticus fabricii; class: Insecta; order: Coleoptera; family: Dytiscidae; **Location:** island: Bali; country: Indonesia; stateProvince: Bali; county: Karangasem; locality: Manggis; verbatimElevation: 40m; locationRemarks: https://goo.gl/maps/rftZoZG1GGk; decimalLatitude: -8.484924; decimalLongitude: 115.528523; **Event:** samplingProtocol: collected with strainer; samplingEffort: 2016-09-10; eventDate: 10.ix.2016; **Record Level:** institutionCode: MZB; collectionCode: Entomology; ownerInstitutionCode: Museum Zoologicum Bogoriense
- **Type status:** Other material. **Occurrence:** recordNumber: BALI_NS_2016_20; recordedBy: Suprayitno; individualCount: 1; lifeStage: adult; **Taxon:** scientificName: Hydaticus fabricii; class: Insecta; order: Coleoptera; family: Dytiscidae; **Location:** island: Bali; country: Indonesia; stateProvince: Bali; county: Karangasem; locality: Jalan Karangasem - Seraya (5); verbatimElevation: 170m; locationRemarks: https://goo.gl/maps/fAT8hjLK4Y82; decimalLatitude: -8.417738; decimalLongitude: 115.688551; **Event:** samplingProtocol: collected with strainer; samplingEffort: 2016-06-16; eventDate: 16.vi.2016; **Record Level:** institutionCode: MZB; collectionCode: Entomology; ownerInstitutionCode: Museum Zoologicum Bogoriense
- **Type status:** Other material. **Occurrence:** recordNumber: BALI_NS_2016_22; recordedBy: Suprayitno; individualCount: 1; lifeStage: adult; **Taxon:** scientificName: Hydaticus fabricii; class: Insecta; order: Coleoptera; family: Dytiscidae; **Location:** island: Bali; country: Indonesia; stateProvince: Bali; county: Gianyar; locality: Batubulan; verbatimElevation: 30m; locationRemarks: http://maps.google.com/?q=-8.623022,115.267418&hl=en&gl=us; decimalLatitude: -8.623022; decimalLongitude: 115.267418; **Event:** samplingProtocol: collected with strainer; samplingEffort: 2016-02-16; eventDate: 16.ii.2016; **Record Level:** institutionCode: MZB; collectionCode: Entomology; ownerInstitutionCode: Museum Zoologicum Bogoriense
- **Type status:** Other material. **Occurrence:** recordNumber: BALI_NS_2016_27; recordedBy: Suprayitno; individualCount: 3; lifeStage: adult; **Taxon:** scientificName: Hydaticus fabricii; class: Insecta; order: Coleoptera; family: Dytiscidae; **Location:** island: Bali; country: Indonesia; stateProvince: Bali; county: Badung; locality: Jl.Tambakbayuh, Ds. Pererenan, Mengwi; verbatimElevation: 30m; locationRemarks: https://goo.gl/maps/nrVaj1pcQ5P2; decimalLatitude: -8.626453; decimalLongitude: 115.135793; **Event:** samplingProtocol: collected with strainer; samplingEffort: 2016-07-22; eventDate: 22.vii.2016; **Record Level:** institutionCode: MZB; collectionCode: Entomology; ownerInstitutionCode: Museum Zoologicum Bogoriense
- **Type status:** Other material. **Occurrence:** recordNumber: BALI_NS_2016_31; recordedBy: Suprayitno; individualCount: 4; lifeStage: adult; **Taxon:** scientificName: Hydaticus fabricii; class: Insecta; order: Coleoptera; family: Dytiscidae; **Location:** island: Bali; country: Indonesia; stateProvince: Bali; county: Gianyar; locality: Jl. Katiklantang, Ds. Singakerta, Ubud; verbatimElevation: 190m; locationRemarks: https://goo.gl/maps/tcZcCJwQrzT2; decimalLatitude: -8.517321; decimalLongitude: 115.251018; **Event:** samplingProtocol: collected with strainer; samplingEffort: 2016-07-06; eventDate: 6.vii.2016; **Record Level:** institutionCode: MZB; collectionCode: Entomology; ownerInstitutionCode: Museum Zoologicum Bogoriense
- **Type status:** Other material. **Occurrence:** recordNumber: BALI_NS_2016_34; recordedBy: Suprayitno; individualCount: 5; lifeStage: adult; **Taxon:** scientificName: Hydaticus fabricii; class: Insecta; order: Coleoptera; family: Dytiscidae; **Location:** island: Bali; country: Indonesia; stateProvince: Bali; county: Badung; locality: Sedang village, Abiansemal; verbatimElevation: 120m; locationRemarks: http://maps.google.com/?q=-8.564203,115.237549&hl=en&gl=us; decimalLatitude: -8.564203; decimalLongitude: 115.237549; **Event:** samplingProtocol: collected with strainer; samplingEffort: 2016-02-29; eventDate: 29.ii.2016; **Record Level:** institutionCode: MZB; collectionCode: Entomology; ownerInstitutionCode: Museum Zoologicum Bogoriense
- **Type status:** Other material. **Occurrence:** recordNumber: BALI_NS_2016_37; recordedBy: Suprayitno; individualCount: 18; lifeStage: adult; **Taxon:** scientificName: Hydaticus fabricii; class: Insecta; order: Coleoptera; family: Dytiscidae; **Location:** island: Bali; country: Indonesia; stateProvince: Bali; county: Klungkung; locality: Tukad Yeh Unda River; verbatimElevation: 6m; locationRemarks: https://goo.gl/maps/fxseqTb5b6N2; decimalLatitude: -8.561778; decimalLongitude: 115.43021; **Event:** samplingProtocol: collected with strainer; samplingEffort: 2016-02-05; eventDate: 5.ii.2016; **Record Level:** institutionCode: MZB; collectionCode: Entomology; ownerInstitutionCode: Museum Zoologicum Bogoriense
- **Type status:** Other material. **Occurrence:** recordNumber: BALI_NS_2016_39; recordedBy: Suprayitno; individualCount: 2; lifeStage: adult; **Taxon:** scientificName: Hydaticus fabricii; class: Insecta; order: Coleoptera; family: Dytiscidae; **Location:** island: Bali; country: Indonesia; stateProvince: Bali; county: Karangasem; locality: Jl. Raya Yehpoh - Manggis; verbatimElevation: 50m; locationRemarks: http://maps.google.com/?q=-8.486125,115.516439&hl=en&gl=us; decimalLatitude: -8.486125; decimalLongitude: 115.516439; **Event:** samplingProtocol: collected with strainer; samplingEffort: 2016-11-05; eventDate: 5.xi.2016; **Record Level:** institutionCode: MZB; collectionCode: Entomology; ownerInstitutionCode: Museum Zoologicum Bogoriense
- **Type status:** Other material. **Occurrence:** recordNumber: BALI_NS_2016_51; recordedBy: Suprayitno; individualCount: 1; lifeStage: adult; **Taxon:** scientificName: Hydaticus fabricii; class: Insecta; order: Coleoptera; family: Dytiscidae; **Location:** island: Bali; country: Indonesia; stateProvince: Bali; county: Gianyar; locality: Jl. Yudistira - Batubulan; verbatimElevation: 40m; locationRemarks: https://goo.gl/maps/sQHVTyGEDkA2; decimalLatitude: -8.624977; decimalLongitude: 115.276241; **Event:** samplingProtocol: collected with strainer; samplingEffort: 2016-12-31; eventDate: 31.xii.2016; **Record Level:** institutionCode: MZB; collectionCode: Entomology; ownerInstitutionCode: Museum Zoologicum Bogoriense
- **Type status:** Other material. **Occurrence:** recordNumber: BALI_NS_2017_55; recordedBy: Suprayitno; individualCount: 2; lifeStage: adult; **Taxon:** scientificName: Hydaticus fabricii; class: Insecta; order: Coleoptera; family: Dytiscidae; **Location:** island: Bali; country: Indonesia; stateProvince: Bali; county: Gianyar; locality: Jl. Katiklantang - Desa Singakerta - Ubud; verbatimElevation: 200m; locationRemarks: https://goo.gl/maps/jpGV7BM7ixN2; decimalLatitude: -8.515147; decimalLongitude: 115.248926; **Event:** samplingProtocol: collected with strainer; samplingEffort: 2017-01-20; eventDate: 20.i.2017; **Record Level:** institutionCode: MZB; collectionCode: Entomology; ownerInstitutionCode: Museum Zoologicum Bogoriense
- **Type status:** Other material. **Occurrence:** recordNumber: BALI_NS_2016_61; recordedBy: Suprayitno; individualCount: 1; lifeStage: adult; **Taxon:** scientificName: Hydaticus fabricii; class: Insecta; order: Coleoptera; family: Dytiscidae; **Location:** island: Bali; country: Indonesia; stateProvince: Bali; county: Karangasem; locality: Unnamed road - Bunutan - Abang; verbatimElevation: 100m; locationRemarks: https://goo.gl/maps/Efa9yguQBB32; decimalLatitude: -8.360504; decimalLongitude: 115.662144; **Event:** samplingProtocol: collected with strainer; samplingEffort: 2016-04-14; eventDate: 14.iv.2016; **Record Level:** institutionCode: MZB; collectionCode: Entomology; ownerInstitutionCode: Museum Zoologicum Bogoriense
- **Type status:** Other material. **Occurrence:** recordNumber: BALI_NS_2017_62; recordedBy: Suprayitno; individualCount: 1; lifeStage: adult; **Taxon:** scientificName: Hydaticus fabricii; class: Insecta; order: Coleoptera; family: Dytiscidae; **Location:** island: Bali; country: Indonesia; stateProvince: Bali; county: Klungkung; locality: Desa Selat, Klungkung, Alam rafting finish point river; verbatimElevation: 240m; locationRemarks: http://maps.google.com/?q=-8.478910,115.410956&hl=en&gl=us; decimalLatitude: -8.47891; decimalLongitude: 115.410956; **Event:** samplingProtocol: collected with strainer; samplingEffort: 2017-04-13; eventDate: 13.iv.2017; **Record Level:** institutionCode: MZB; collectionCode: Entomology; ownerInstitutionCode: Museum Zoologicum Bogoriense
- **Type status:** Other material. **Occurrence:** recordNumber: BA3; recordedBy: Hendrich and Balke; individualCount: 4; lifeStage: adult; **Taxon:** scientificName: Hydaticus fabricii; class: Insecta; order: Coleoptera; family: Dytiscidae; **Location:** island: Bali; country: Indonesia; stateProvince: Bali; county: Gianyar; locality: Ubud; verbatimElevation: 180m; decimalLatitude: -8.516324; decimalLongitude: 115.257336; **Event:** samplingProtocol: collected with strainer; samplingEffort: 1990-08-26; eventDate: 26.viii.1990; **Record Level:** institutionCode: ZSM etc; collectionCode: Entomology; ownerInstitutionCode: Zooligical State Collection Munich

#### Distribution in Bali

See Fig. 21.

#### Geographic range outside Bali

India, SE Asia, Sunda islands as far east as Sumba (Wewalka 1979).

#### Ecology

**Figure 22.**
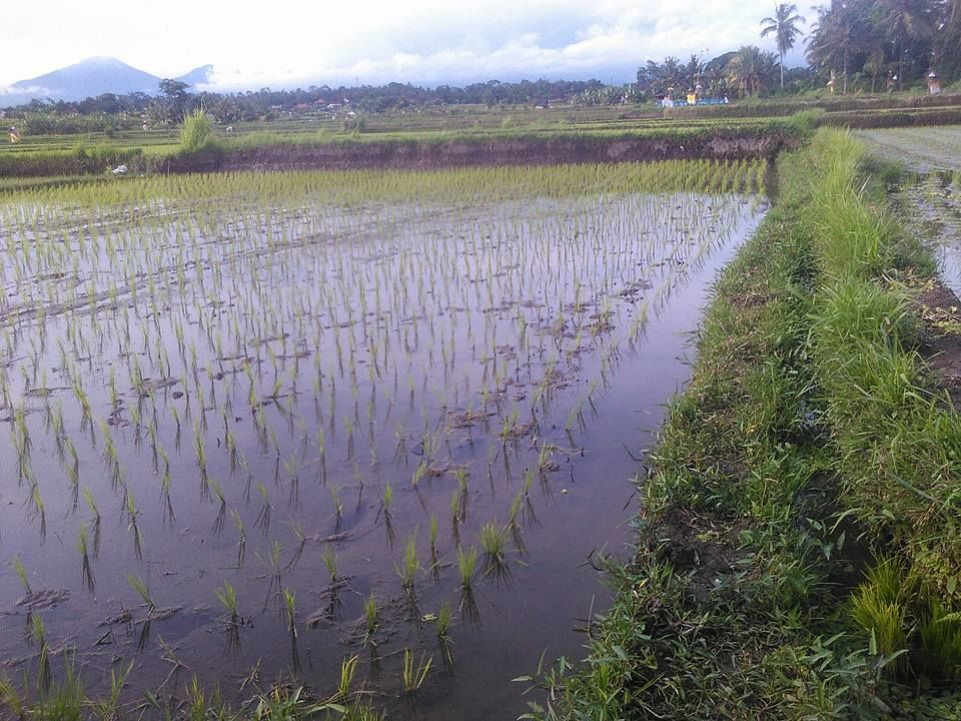
Collecting site BALI_NS_2016_55.

The species is an inhabitatnt of stagnant water habitats, often in rice paddies (Fig. 22).

### Hydaticus luczonicus

Aubé, 1838

Hydaticus luczonicus Aubé 1838: 179; Vazirani 1969: 262; Nilsson and Hájek 2017: 93.

#### Materials

- **Type status:** Other material. **Occurrence:** recordNumber: BALI_NS_2016_18; recordedBy: Suprayitno; individualCount: 2; **Taxon:** scientificName: Hydaticus luczonicus; class: Insecta; order: Coleoptera; family: Dytiscidae; **Location:** island: Bali; country: Indonesia; stateProvince: Bali; county: Karangasem; locality: Jalan Karangasem - Seraya (2); verbatimElevation: 270m; locationRemarks: https://goo.gl/maps/MRMj83F9AAz; decimalLatitude: -8.421717; decimalLongitude: 115.669085; **Event:** samplingProtocol: collected with strainer; eventDate: 4.vi.2016-06-04; **Record Level:** institutionCode: MZB; collectionCode: Entomology; ownerInstitutionCode: Museum Zoologicum Bogoriense

#### Distribution in Bali

See Fig. 23.

#### Geographic range outside Bali

Philippines, SE Asia. First record for Bali.

#### Ecology

**Figure 24.**
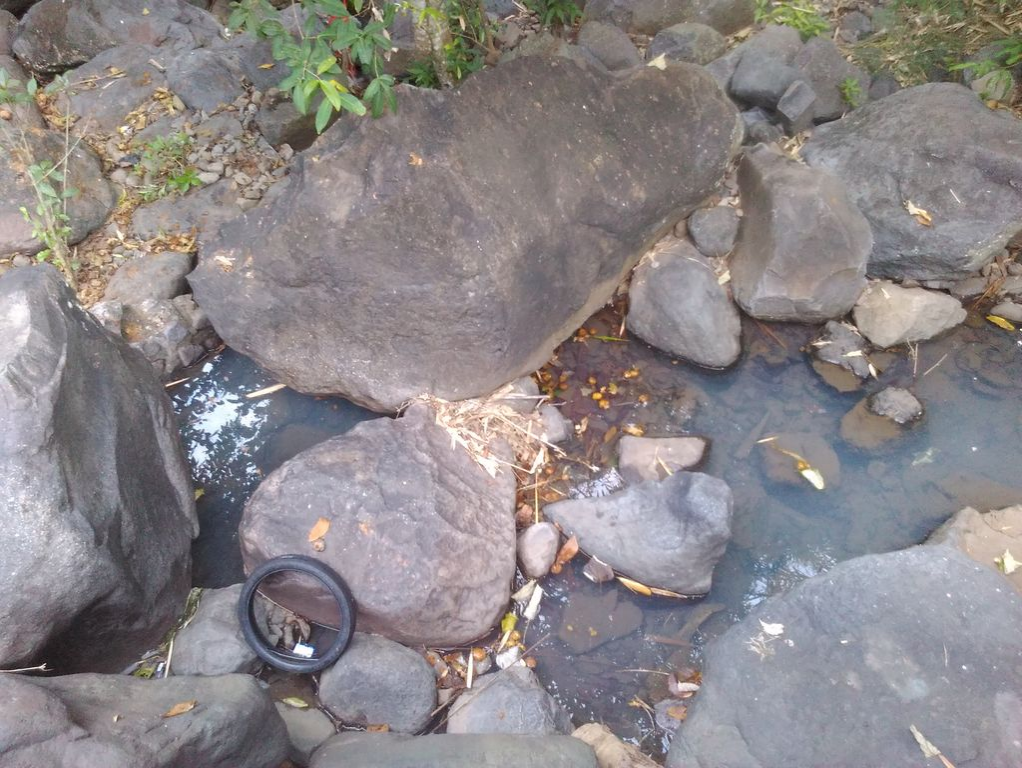
Collecting site BALI_NS_2016_18.

The species occupies stagnant water habitats. In Bali, it was collected in rest pools in a streambed on volcanic rock (Fig. 24).

### Hydaticus pacificus

Aubé, 1838

https://www.facebook.com/Baliwaterbeetles/photos/a.363732503750926.1073741835.359144747543035/363733343750842/?type=3&theater

Hydaticus pacificus Aubé 1838: 177; Wewalka 2016: 11; Nilsson and Hájek 2017: 94.

#### Materials

- **Type status:** Other material. **Occurrence:** recordNumber: BALI_NS_2016_16; recordedBy: Suprayitno; individualCount: 2; lifeStage: adult; **Taxon:** scientificName: Hydaticus pacificus; class: Insecta; order: Coleoptera; family: Dytiscidae; **Location:** island: Bali; country: Indonesia; stateProvince: Bali; county: Karangasem; locality: Jalan Karangasem - Seraya; verbatimElevation: 350m; locationRemarks: https://goo.gl/maps/ftkQnXGqTjC2; decimalLatitude: -8.410798; decimalLongitude: 115.6815772; **Event:** samplingProtocol: collected with strainer; eventDate: 2016-06-15; **Record Level:** institutionCode: MZB; collectionCode: Entomology; ownerInstitutionCode: Museum Zoologicum Bogoriense
- **Type status:** Other material. **Occurrence:** recordNumber: BALI_NS_2016_17; recordedBy: Suprayitno; individualCount: 4; lifeStage: adult; **Taxon:** scientificName: Hydaticus pacificus; class: Insecta; order: Coleoptera; family: Dytiscidae; **Location:** island: Bali; country: Indonesia; stateProvince: Bali; county: Karangasem; locality: Jalan Karangasem - Seraya; verbatimElevation: 250m; locationRemarks: https://goo.gl/maps/vp4fVVnqXsy; decimalLatitude: -8.426531; decimalLongitude: 115.661005; **Event:** samplingProtocol: collected with strainer; eventDate: 2016-06-02; **Record Level:** institutionCode: MZB; collectionCode: Entomology; ownerInstitutionCode: Museum Zoologicum Bogoriense
- **Type status:** Other material. **Occurrence:** recordNumber: BALI_NS_2016_18; recordedBy: Suprayitno; individualCount: 1; lifeStage: adult; **Taxon:** scientificName: Hydaticus pacificus; class: Insecta; order: Coleoptera; family: Dytiscidae; **Location:** island: Bali; country: Indonesia; stateProvince: Bali; county: Karangasem; locality: Jalan Karangasem - Seraya; verbatimElevation: 270m; locationRemarks: https://goo.gl/maps/MRMj83F9AAz; decimalLatitude: -8.421717; decimalLongitude: 115.669085; **Event:** samplingProtocol: collected with strainer; eventDate: 2016-06-04; **Record Level:** institutionCode: MZB; collectionCode: Entomology; ownerInstitutionCode: Museum Zoologicum Bogoriense
- **Type status:** Other material. **Occurrence:** recordNumber: BALI_NS_2016_19; recordedBy: Suprayitno; individualCount: 3; lifeStage: adult; **Taxon:** scientificName: Hydaticus pacificus; class: Insecta; order: Coleoptera; family: Dytiscidae; **Location:** island: Bali; country: Indonesia; stateProvince: Bali; county: Karangasem; locality: Jalan Karangasem - Seraya; verbatimElevation: 270m; locationRemarks: https://goo.gl/maps/GJpzEosySy12; decimalLatitude: -8.421574; decimalLongitude: 115.671857; **Event:** samplingProtocol: collected with strainer; eventDate: 2016-06-05; **Record Level:** institutionCode: MZB; collectionCode: Entomology; ownerInstitutionCode: Museum Zoologicum Bogoriense
- **Type status:** Other material. **Occurrence:** recordNumber: BALI_NS_2016_20; recordedBy: Suprayitno; individualCount: 28; lifeStage: adult; **Taxon:** scientificName: Hydaticus pacificus; class: Insecta; order: Coleoptera; family: Dytiscidae; **Location:** island: Bali; country: Indonesia; stateProvince: Bali; county: Karangasem; locality: Jalan Karangasem - Seraya; verbatimElevation: 170m; locationRemarks: https://goo.gl/maps/fAT8hjLK4Y82; decimalLatitude: -8.417738; decimalLongitude: 115.688551; **Event:** samplingProtocol: collected with strainer; eventDate: 2016-06-16; **Record Level:** institutionCode: MZB; collectionCode: Entomology; ownerInstitutionCode: Museum Zoologicum Bogoriense
- **Type status:** Other material. **Occurrence:** recordNumber: BALI_NS_2016_23; recordedBy: Suprayitno; individualCount: 2; lifeStage: adult; **Taxon:** scientificName: Hydaticus pacificus; class: Insecta; order: Coleoptera; family: Dytiscidae; **Location:** island: Bali; country: Indonesia; stateProvince: Bali; county: Badung; locality: Desa Ungasan near Pecatu; verbatimElevation: 140m; locationRemarks: https://goo.gl/maps/p64cgybQRDF2; decimalLatitude: -8.840126; decimalLongitude: 115.144003; **Event:** samplingProtocol: collected with strainer; eventDate: 2016-06-20; **Record Level:** institutionCode: MZB; collectionCode: Entomology; ownerInstitutionCode: Museum Zoologicum Bogoriense

#### Distribution in Bali

See Fig. 25.

#### Geographic range outside Bali

SE Asia, Sunda islands as far east as Tanimbar; not on Borneo or Sulawesi. This species was first recorded for Bali by Wewalka (2016) based on photography we presented on FaceBook.

#### Ecology

**Figure 26.**
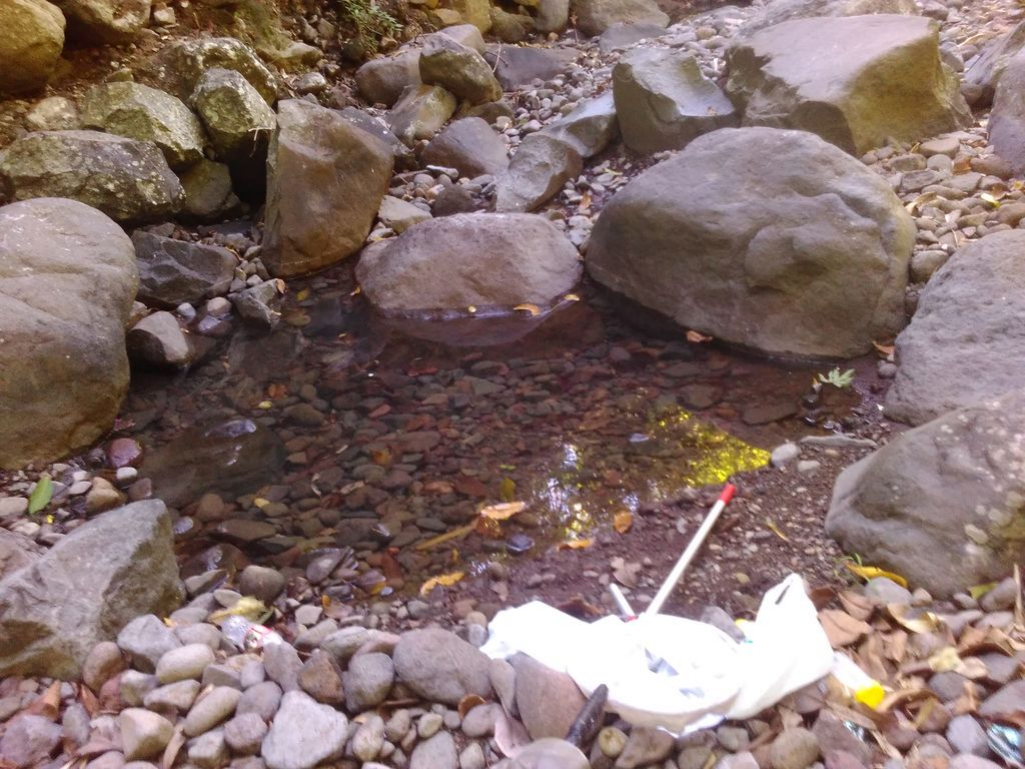
Collecting site BALI_NS_2016_17.

**Figure 27.**
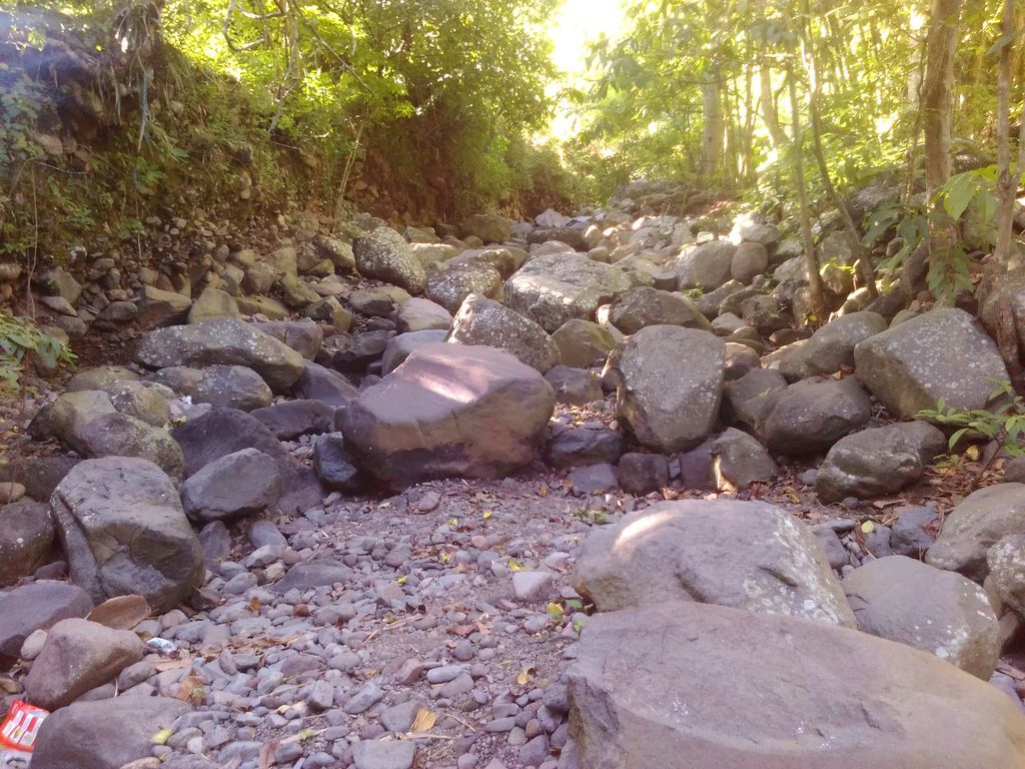
Collecting site BALI_NS_2016_17.

**Figure 28.**
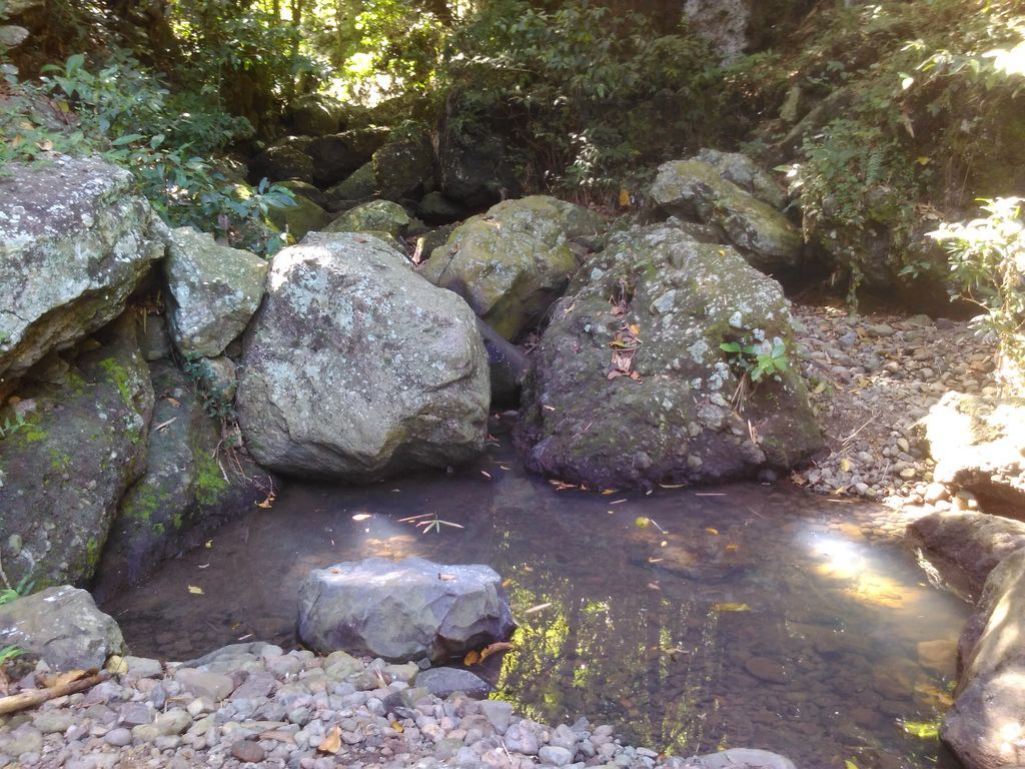
Collecting site BALI_NS_2016_19.

**Figure 29.**
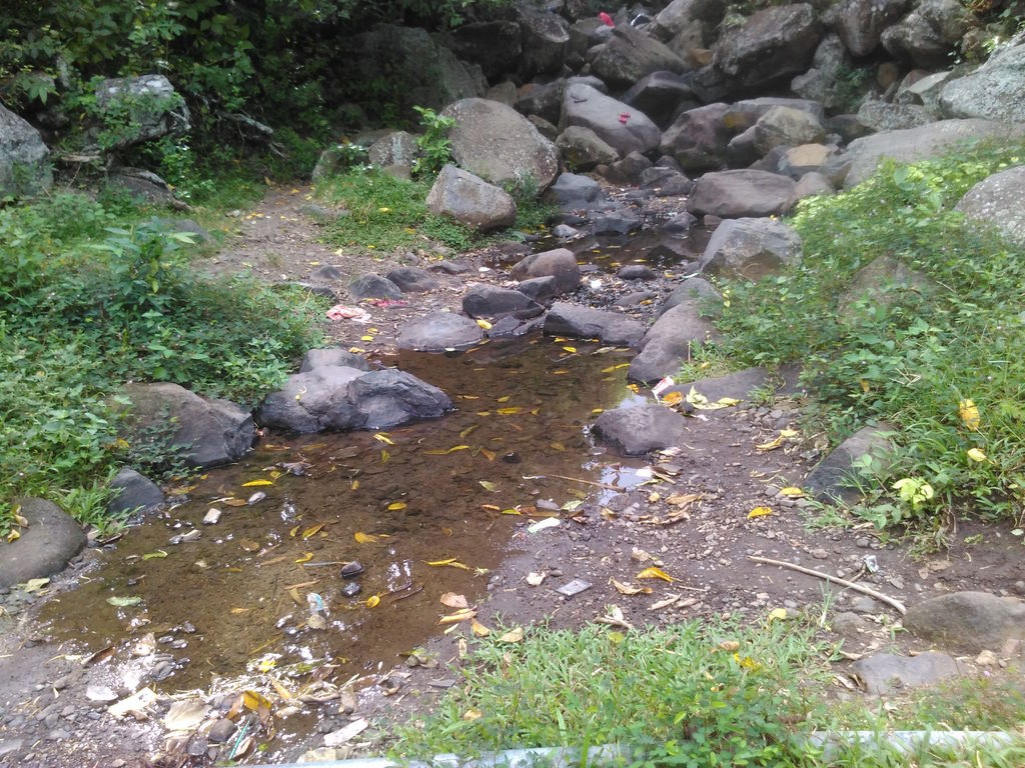
Collecting site BALI_NS_2016_16.

The species occupies stagnant water bodies. In Bali, it was often found in pools of intermittent streams (Figs 26, 27, 28, 29).

### Microdytes elgae

Hendrich, Balke & Wewalka, 1995

Microdytes elgae Hendrich and Balke 1995: 42; Aubé 1838: 177; Nilsson and Hájek 2017: 202.

#### Materials

- **Type status:** Other material. **Occurrence:** recordNumber: BALI_NS_2016_07; recordedBy: Suprayitno; individualCount: 7; lifeStage: adult; **Taxon:** scientificName: Microdytes elgae; class: Insecta; order: Coleoptera; family: Dytiscidae; **Location:** island: Bali; country: Indonesia; stateProvince: Bali; county: Tabanan; locality: Jl. Raya Apuan Senganan - Baturiti; verbatimElevation: 600m; locationRemarks: https://goo.gl/maps/DwRBDdgGPwp; decimalLatitude: -8.362083; decimalLongitude: 115.180111; **Event:** samplingProtocol: collected with strainer; eventDate: 2016-09-04; **Record Level:** institutionCode: MZB; collectionCode: Entomology; ownerInstitutionCode: Museum Zoologicum Bogoriense
- **Type status:** Other material. **Occurrence:** recordNumber: BALI_NS_2016_35; recordedBy: Suprayitno; individualCount: 1; lifeStage: adult; **Taxon:** scientificName: Microdytes elgae; class: Insecta; order: Coleoptera; family: Dytiscidae; **Location:** island: Bali; country: Indonesia; stateProvince: Bali; county: Buleleng; locality: Ds. Selat, Sukasada; verbatimElevation: 180m; locationRemarks: https://goo.gl/maps/DVC4v6KZQ2R2; decimalLatitude: -8.174688; decimalLongitude: 115.066921; **Event:** samplingProtocol: collected with strainer; eventDate: 2016-05-31; **Record Level:** institutionCode: MZB; collectionCode: Entomology; ownerInstitutionCode: Museum Zoologicum Bogoriense
- **Type status:** Other material. **Occurrence:** recordNumber: BALI_NS_2017_56; recordedBy: Suprayitno; individualCount: 22; lifeStage: adult; **Taxon:** scientificName: Microdytes elgae; class: Insecta; order: Coleoptera; family: Dytiscidae; **Location:** island: Bali; country: Indonesia; stateProvince: Bali; county: Tabanan; locality: Jl. Bantas - Pajahan - Pupuan; verbatimElevation: 500m; locationRemarks: https://goo.gl/maps/2JtWBirCLHR2; decimalLatitude: -8.345852; decimalLongitude: 114.994454; **Event:** samplingProtocol: collected with strainer; eventDate: 2017-01-26; **Record Level:** institutionCode: MZB; collectionCode: Entomology; ownerInstitutionCode: Museum Zoologicum Bogoriense
- **Type status:** Other material. **Occurrence:** recordNumber: BA5; recordedBy: Hendrich & Balke; individualCount: 2; lifeStage: adult; **Taxon:** scientificName: Microdytes elgae; class: Insecta; order: Coleoptera; family: Dytiscidae; **Location:** island: Bali; country: Indonesia; stateProvince: Bali; locality: Bedugul, Candi Kuning; verbatimElevation: 1320m; decimalLatitude: -8.253661; decimalLongitude: 115.173608; **Event:** samplingProtocol: collected with strainer; eventDate: 1990-08-27 and 1991-07-11; **Record Level:** institutionCode: ZSM etc; collectionCode: Entomology; datasetName: Zooligical State Collection Munich; ownerInstitutionCode: Zooligical State Collection Munich; basisOfRecord: Entomology; informationWithheld: Zooligical State Collection Munich

#### Distribution in Bali

See Fig. 30.

#### Geographic range outside Bali

Widespread through the SE Paleartic region and SE Asia (Bhutan, Indonesia [Bali, Lombok, Kalimantan], Malaysia [Peninsula, Sarawak], Singapore) (Balke et al. 1999, Hendrich et al. 2004).

#### Ecology

**Figure 31.**
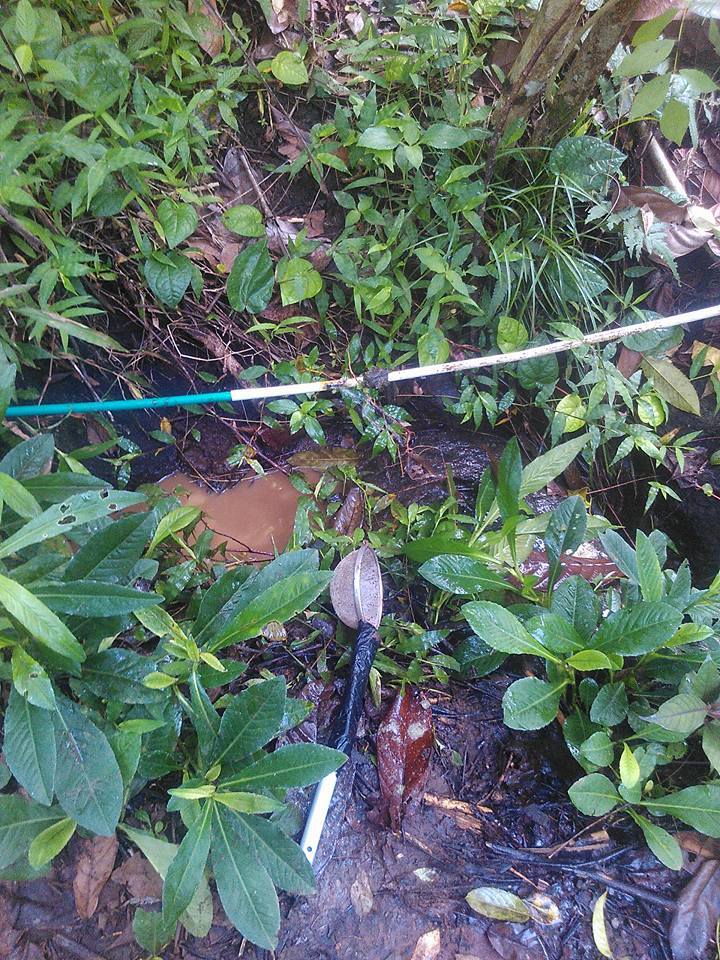
Collecting site BALI_NS_2016_56.

**Figure 32.**
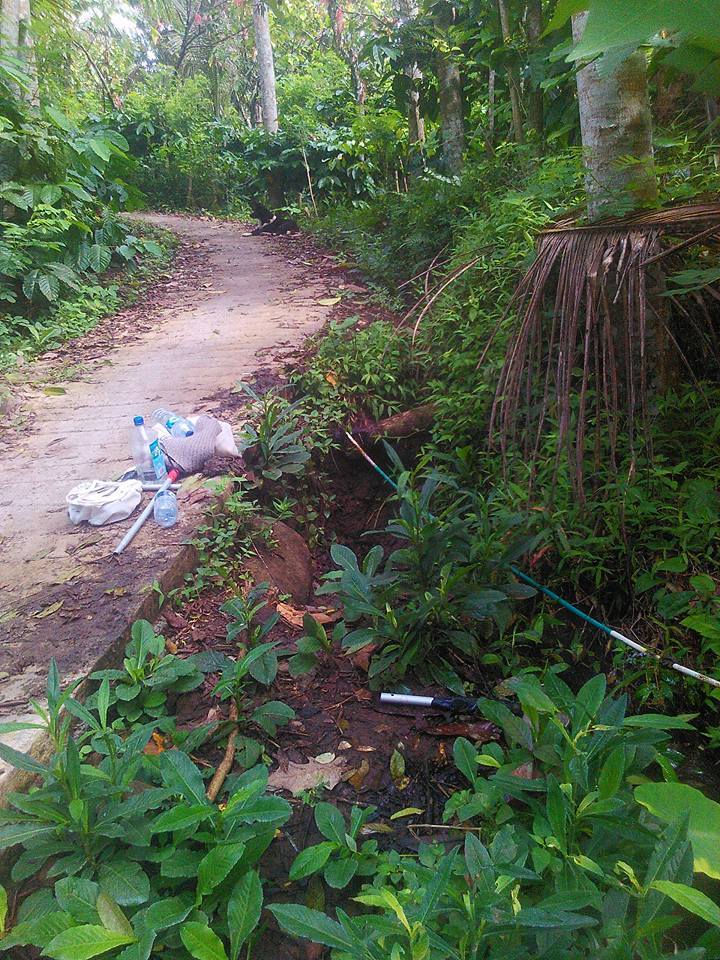
Collecting site BALI_NS_2016_56.

*Microdytes elgae* inhabits small shaded or semishaded helocrenes as well as small streams in shaded forest environments. The beetles usually hide beneath leaves and in the gravel under only a very thin film of water (Hendrich et al. 2004) (Figs 31, 32).

### Sandracottus hunteri

(Crotch, 1872)

https://www.facebook.com/Baliwaterbeetles/photos/a.363732503750926.1073741835.359144747543035/363732707084239/?type=3&theater

Sandracottus hunteri Crotch 1872: 205; Hendrich and Balke 1995: 47; Nilsson and Hájek 2017: 85.
Sandracottus hunteri Note, this species was to date treated under the name *S. mixtus* (Blanchard, 1843). However, this name is not applicable to the species treated here, with the available name being *Hydaticus hunteri* Crotch, 1872 (a taxonomic clarification will be presented by Hendrich, in prep.).

#### Materials

- **Type status:** Other material. **Occurrence:** recordNumber: BALI_NS_2016_16; recordedBy: Suprayitno; individualCount: 1; lifeStage: adult; **Taxon:** scientificName: Sandracottus hunteri; class: Insecta; order: Coleoptera; family: Dytiscidae; **Location:** island: Bali; country: Indonesia; stateProvince: Bali; county: Karangasem; locality: Jalan Karangasem - Seraya (4); verbatimElevation: 300m; locationRemarks: https://goo.gl/maps/ftkQnXGqTjC2; decimalLatitude: -8.410798; decimalLongitude: 115.6815772; **Event:** samplingProtocol: collected with strainer; eventDate: 2016-08-21; **Record Level:** institutionCode: MZB; collectionCode: Entomology; ownerInstitutionCode: Museum Zoologicum Bogoriense
- **Type status:** Other material. **Occurrence:** recordNumber: BALI_NS_2016_17; recordedBy: Suprayitno; individualCount: 1; lifeStage: adult; **Taxon:** scientificName: Sandracottus hunteri; class: Insecta; order: Coleoptera; family: Dytiscidae; **Location:** island: Bali; country: Indonesia; stateProvince: Bali; county: Karangasem; locality: Jalan Karangasem - Seraya (1); verbatimElevation: 250m; locationRemarks: https://goo.gl/maps/vp4fVVnqXsy; decimalLatitude: -8.426531; decimalLongitude: 115.661005; **Event:** samplingProtocol: collected with strainer; eventDate: 2016-07-02; **Record Level:** institutionCode: MZB; collectionCode: Entomology; ownerInstitutionCode: Museum Zoologicum Bogoriense
- **Type status:** Other material. **Occurrence:** recordNumber: BALI_NS_2016_19; recordedBy: Suprayitno; individualCount: 2; lifeStage: adult; **Taxon:** scientificName: Sandracottus hunteri; class: Insecta; order: Coleoptera; family: Dytiscidae; **Location:** island: Bali; country: Indonesia; stateProvince: Bali; county: Karangasem; locality: Jalan Karangasem - Seraya (3); verbatimElevation: 260m; locationRemarks: https://goo.gl/maps/GJpzEosySy12; decimalLatitude: -8.421574; decimalLongitude: 115.671857; **Event:** samplingProtocol: collected with strainer; eventDate: 2016-07-05; **Record Level:** institutionCode: MZB; collectionCode: Entomology; ownerInstitutionCode: Museum Zoologicum Bogoriense
- **Type status:** Other material. **Occurrence:** recordNumber: BALI_NS_2016_23; recordedBy: Suprayitno; individualCount: 2; lifeStage: adult; **Taxon:** scientificName: Sandracottus hunteri; class: Insecta; order: Coleoptera; family: Dytiscidae; **Location:** island: Bali; country: Indonesia; stateProvince: Bali; county: Badung; locality: Desa Ungasan near Pecatu; verbatimElevation: 140m; locationRemarks: https://goo.gl/maps/p64cgybQRDF2; decimalLatitude: -8.840126; decimalLongitude: 115.144003; **Event:** samplingProtocol: collected with strainer; eventDate: 2016-06-20; **Record Level:** institutionCode: MZB; collectionCode: Entomology; ownerInstitutionCode: Museum Zoologicum Bogoriense
- **Type status:** Other material. **Occurrence:** recordNumber: BA8; recordedBy: Hendrich & Balke; individualCount: 1; lifeStage: adult; **Taxon:** scientificName: Sandracottus hunteri; class: Insecta; order: Coleoptera; family: Dytiscidae; **Location:** island: Bali; country: Indonesia; stateProvince: Bali; locality: Bedugul, Candi Kuning; verbatimElevation: 1320m; decimalLatitude: -8.253661; decimalLongitude: 115.173608; **Event:** samplingProtocol: dipnet; eventDate: 1991-07-11; **Record Level:** institutionCode: ZSM etc; collectionCode: Entomology; datasetName: Zooligical State Collection Munich; ownerInstitutionCode: Zooligical State Collection Munich; basisOfRecord: Entomology; informationWithheld: Zooligical State Collection Munich

#### Distribution in Bali

See Fig. 33.

#### Geographic range outside Bali

Oriental Region.

#### Ecology

Pools in intermittend streams (Figs 26, 28, 29).

## Discussion

The workflow described here has proven useful to connect a highly motivated citizen scientist to expert taxonomists and museum curators. Online information exchange and training is leading to the formation of a parataxonomist, and has led to the enhancement of a national depository. We suggest that this approach would also be very beneficial for researchers at universities in less connected areas, such as ecologists and zoologists, who are frequently confronted with the need to identify species in the course of their work, but are often essentially operating at the same level of taxonomic expertise as an interested amateur. In addition to enabling these researchers to publish higher quality papers, the formation of links to national depositories would add sustainability to biodiversity research where it is most needed. Our approach can also aid in the formation of national as well as international networks using a technology that is fast, readily available and easy to use.

Here, we have presented new distributional data on the diving beetle fauna of Bali. Two new records for the island raise the total number of Dytiscidae species known from Bali to 34. In the future, it is our goal to compile a comprehensive faunistic review of the Balinese fauna using our approach.

## Supplementary Material

XML Treatment for Allopachria quadripustulata

XML Treatment for Cybister tripunctatus temnenkii

XML Treatment for Eretes griseus

XML Treatment for Hydaticus bipunctatus conjungens

XML Treatment for Hydaticus fabricii

XML Treatment for Hydaticus luczonicus

XML Treatment for Hydaticus pacificus

XML Treatment for Microdytes elgae

XML Treatment for Sandracottus hunteri

## Figures and Tables

**Figure 13.**
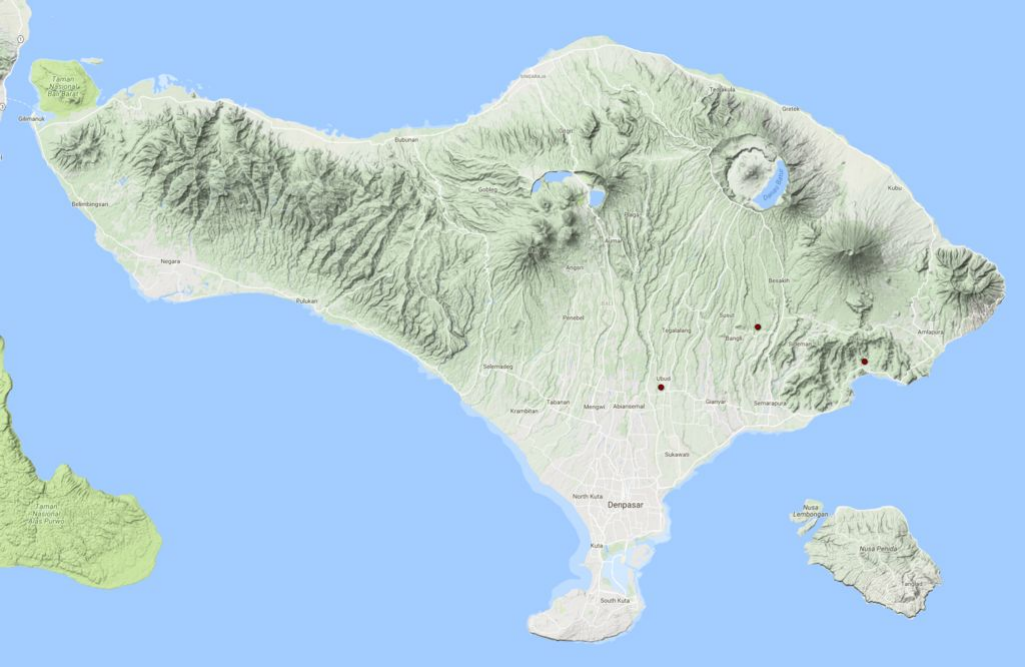
*Allopachria quadripustulata* Distribution in Bali.

**Figure 15.**
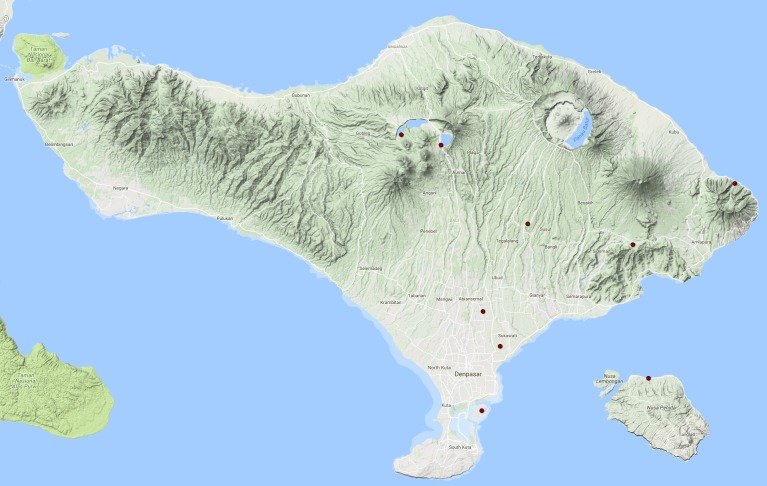
*Cybister tripunctatus temnenkii* Distribution in Bali.

**Figure 16.**
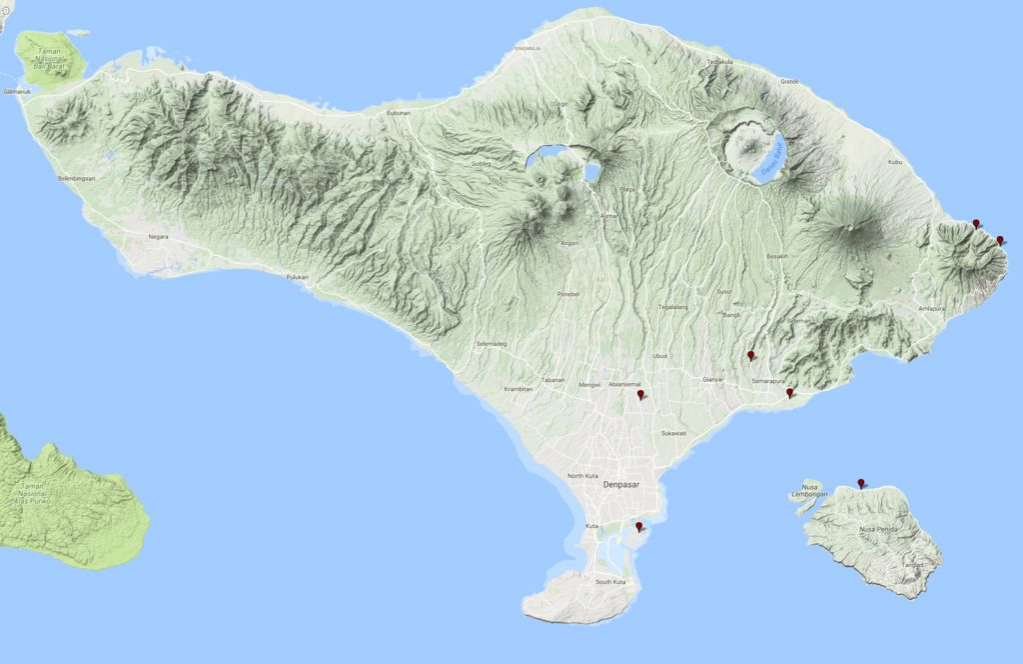
*Eretes griseus* Distribution in Bali.

**Figure 18.**
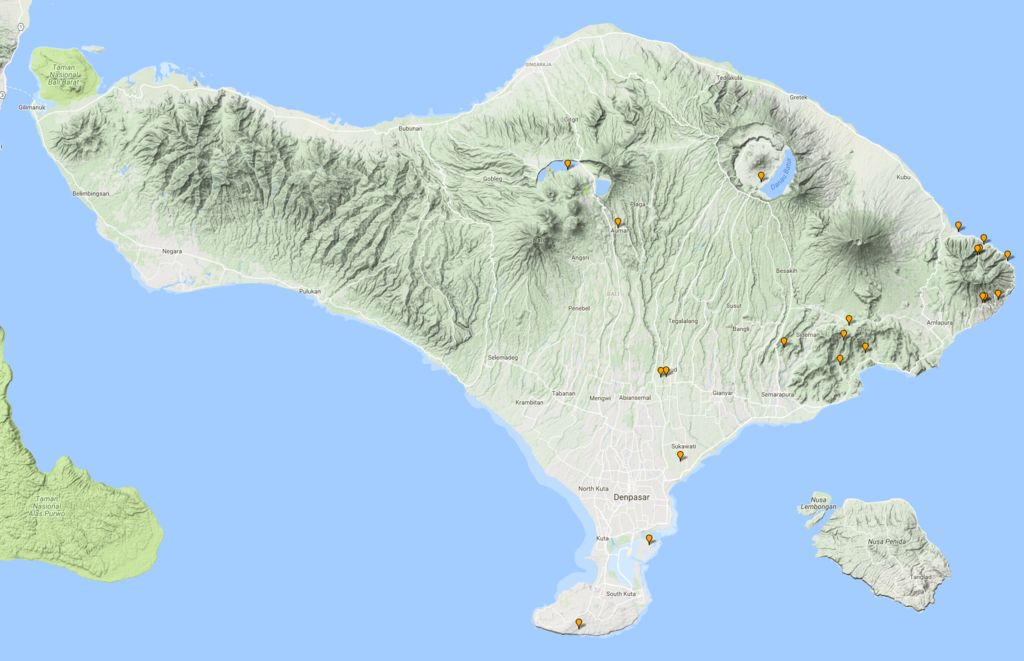
*Hydaticus bipunctatus conjungens* Distribution in Bali.

**Figure 21.**
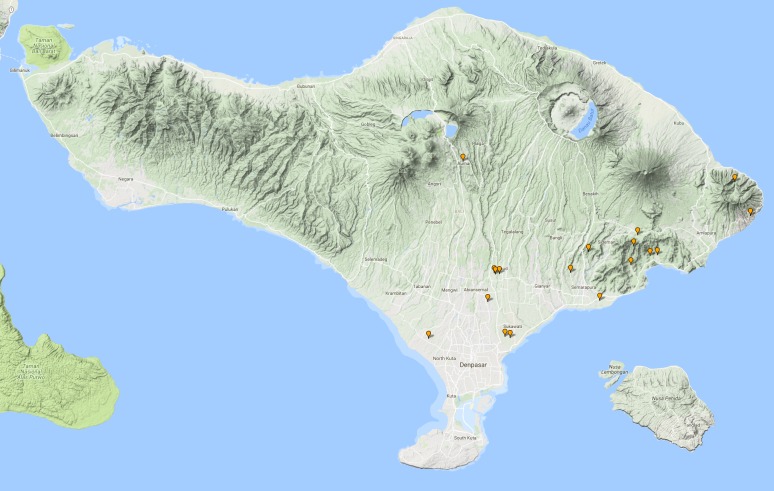
*Hydaticus fabriciii* Distribution in Bali.

**Figure 23.**
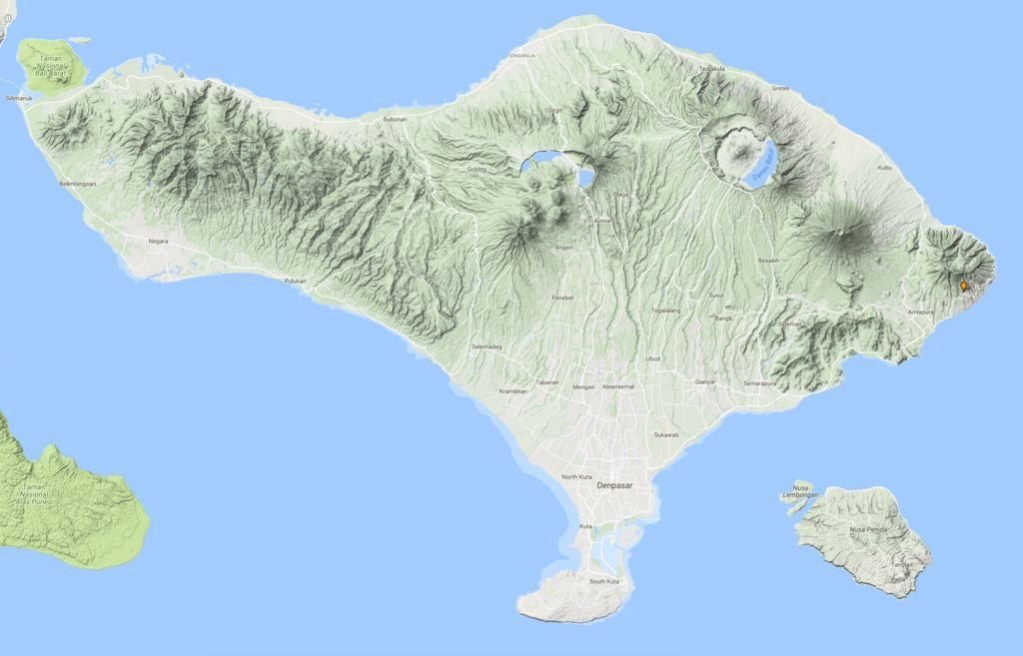
*Hydaticus luczonicus* Distribution in Bali.

**Figure 25.**
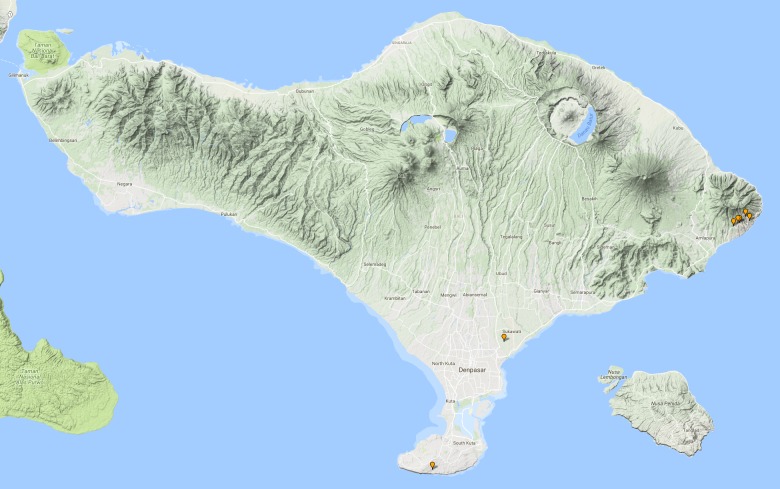
*Hydaticus pacificus* Distribution in Bali.

**Figure 30.**
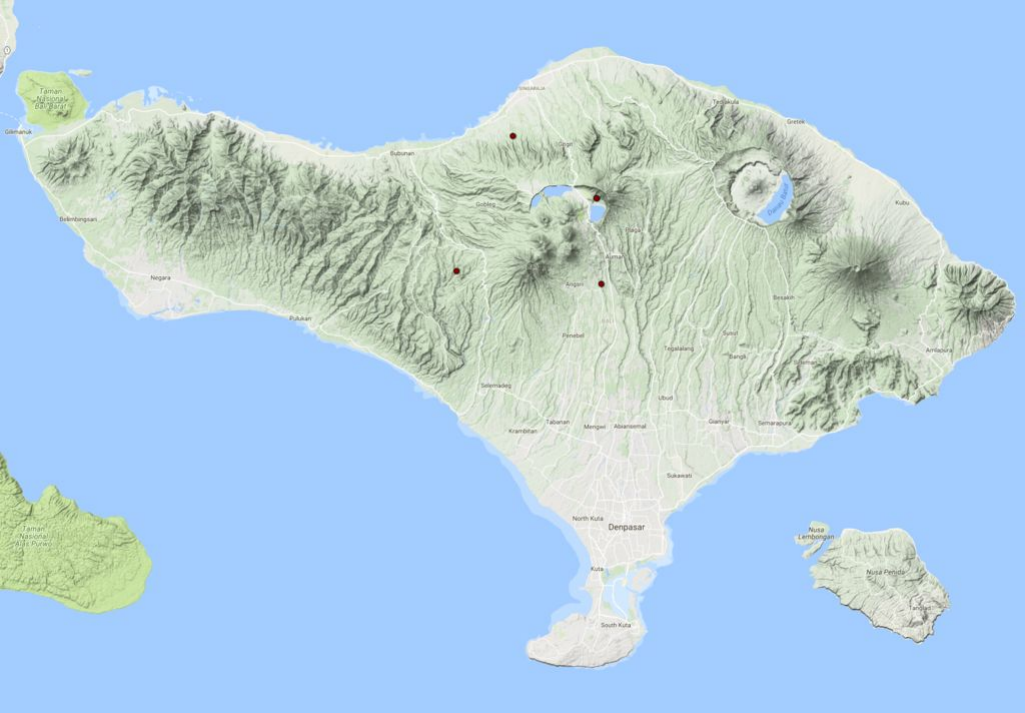
*Microdytes elgae* Distribution in Bali.

**Figure 33.**
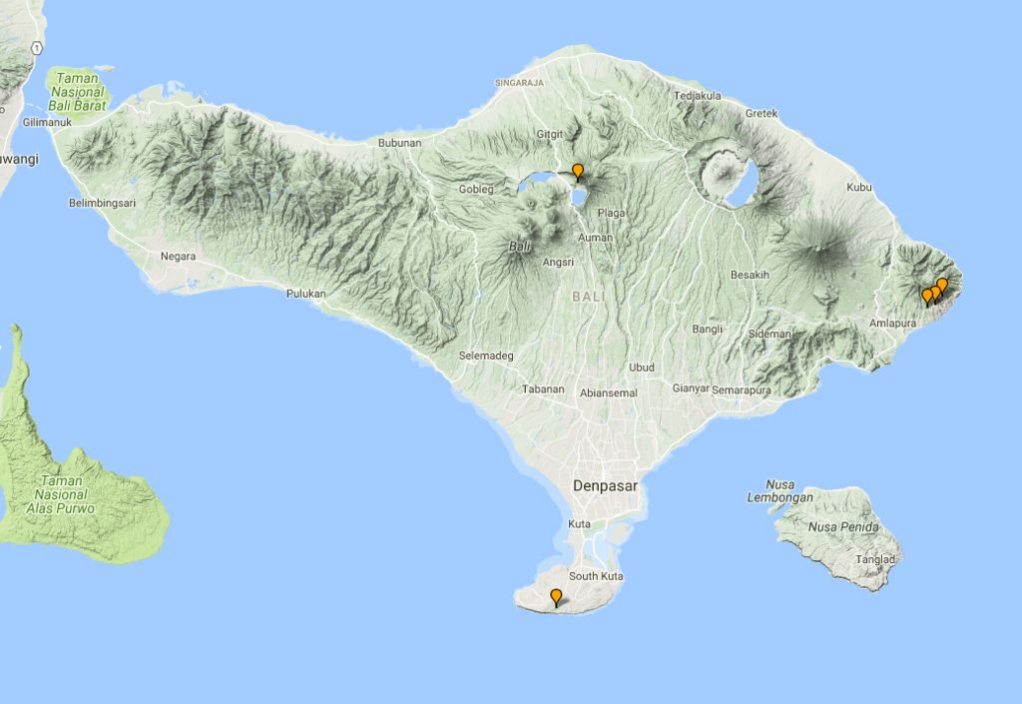
*Sandracottus hunteri* Distribution in Bali.
